# Reactive Secondary Sequence Oxidative Pathology Polymer Model and Antioxidant Tests

**DOI:** 10.9734/IRJPAC/2012/2104

**Published:** 2012

**Authors:** Richard C. Petersen

**Affiliations:** 1 University of Alabama at Birmingham, SDB 539, 1919 7^th^ Avenue South, Biomaterials and Biomedical Engineering, Birmingham AL 35294, USA

**Keywords:** Thermoset polymer, unsaturated lipid, hydroquinone, vitamin A, vitamin E

## Abstract

**Aims:**

To provide common Organic Chemistry/Polymer Science thermoset free-radical crosslinking Sciences for Medical understanding and also present research findings for several common vitamins/antioxidants with a new class of drugs known as free-radical inhibitors.

**Study Design:**

Peroxide/Fenton transition-metal redox couples that generate free radicals were combined with unsaturated lipid oils to demonstrate thermoset-polymer chain growth by crosslinking with the α-β-unsaturated aldehyde acrolein into rubbery/adhesive solids. Further, Vitamin A and beta carotene were similarly studied for crosslink pathological potential. Also, free-radical inhibitor hydroquinone was compared for antioxidant capability with Vitamin E.

**Place and Duration of Study:**

Department of Materials Science and Engineering and Department of Biomaterials, University of Alabama at Birmingham, between June 2005 and August 2012.

**Methodology:**

Observations were recorded for Fenton free-radical crosslinking of unsaturated lipids and vitamin A/beta carotene by photography further with weight measurements and percent-shrinkage testing directly related to covalent crosslinking of unsaturated lipids recorded over time with different concentrations of acrolein. Also, hydroquinone and vitamin E were compared at concentrations from 0.0–7.3wt% as antioxidants for reductions in percent-shrinkage measurements, n = 5.

**Results:**

Unsaturated lipid oils responded to Fenton thermoset-polymer reactive secondary sequence reactions only by acrolein with crosslinking into rubbery-type solids and different non-solid gluey products. Further, molecular oxygen crosslinking was demonstrated with lipid peroxidation and acrolein at specially identified margins. By peroxide/Fenton free-radical testing, both vitamin A and beta-carotene demonstrated possible pathology chemistry for chain-growth crosslinking. During lipid/acrolein testing over a 50 hour time period at 7.3wt% antioxidants, hydroquinone significantly reduced percent shrinkage greatly compared to the standard antioxidant vitamin E, %shrinkage at 11.6 ±1.3 for hydroquinone and 27.8 ±2.2 for vitamin E, *P* = .001.

**Conclusion:**

Free radicals crosslinked unsaturated lipid fatty acids into thermoset polymers through Fenton reactions when combined with acrolein. Further, hydroquinone was a superior antioxidant to vitamin E.

## 1. INTRODUCTION

### 1.1 Organic Chemistry/Polymer Science Background to Atherosclerosis and other Pathologies

**Fig. 1:**
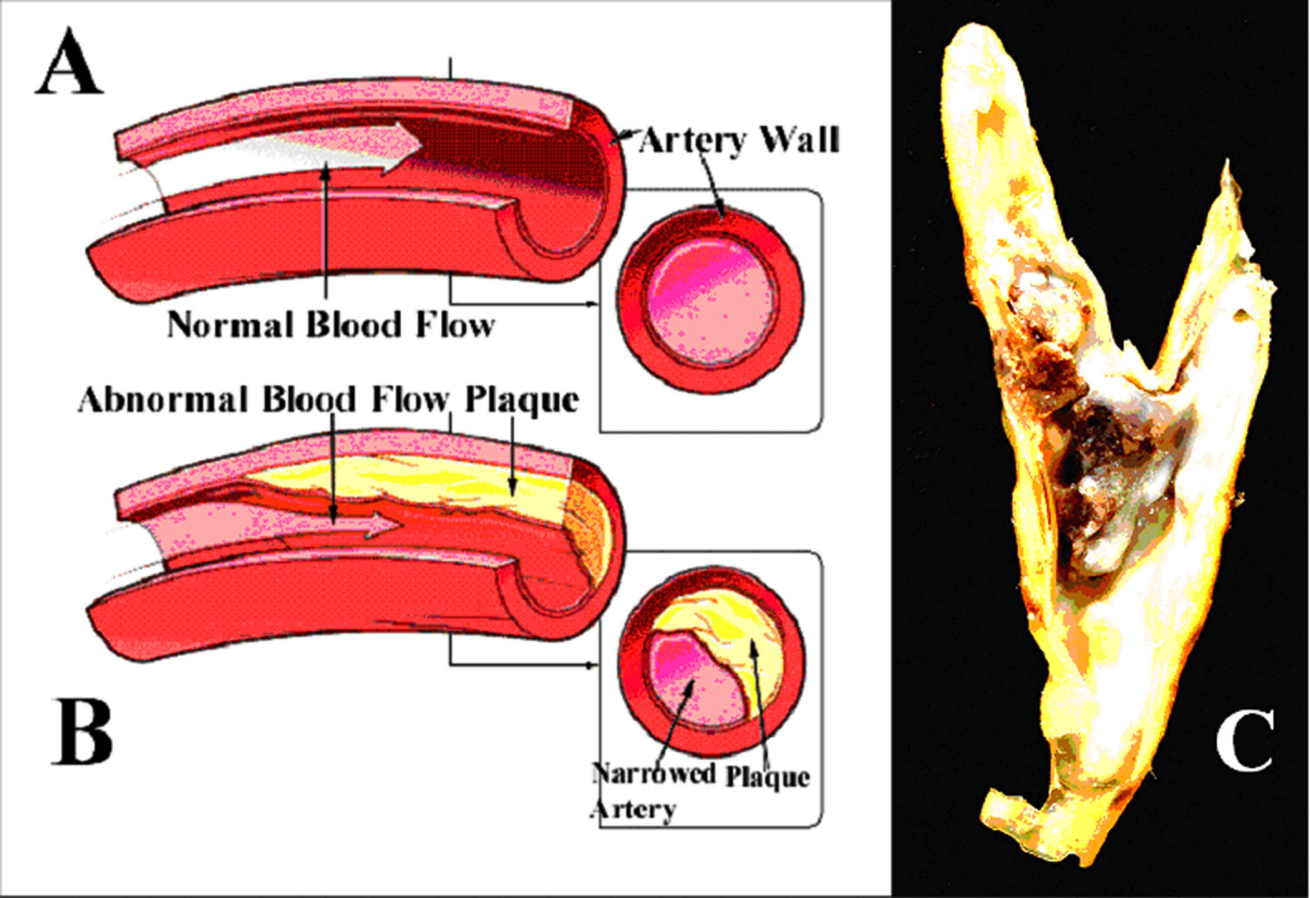
Atherosclerosis: (A) Normal blood flow (B) Abnormal blood flow with lipid-rich plaque (C) Atherosclerotic plaque from vascular tissue that was surgically removed at the bifurcation of the common into the internal and external carotid arteries (A and B With permission National Institutes of Health/Department of Health and Human Services)

New insights into many medical pathological conditions may be discovered by studying free radicals through a fundamental Organic Chemistry approach using Polymer Science. Basic Organic Chemistry knowledge toward understanding free-radical pathology includes the fact that covalent structure imparts general atomic-bond permanency based on stability of the bond linking two atoms together [1–3]. By similar structure-based chemistry, increasing covalent bonding measured by molecular weight increases polymer stability from degradation [4]. Further, polymer protein oriented structures evaluated as biological fiber produce insoluble proteins for tissue strength and elasticity compared to water-soluble amorphous globular proteins with much lower covalent bonding density [1, 4, 5]. Subsequent covalent structure characterized by potential insolubility [1–4] is thus manifested by persistence in particular concern through pathology identified with atherosclerotic dense lipid-rich core plaque in the vasculature with ensuing ischemic diminished blood flow [2, 3, 5, 6]. Atherosclerosis or “hardening of the arteries” is a major Medical problem then that represents widespread pathology with associated high ischemic-related mortality rates [3, 5, 6–8]. In fact, complications from coronary atherosclerotic disease represent the leading cause of death in the Western world since 1990 projected now to 2020 [7, 9, 10], and chief cause at 34.4% of the total deaths in the United States of America [8] with 31% of the total deaths worldwide [6]. Although mortality rates in the United States were once closer to 50% for coronary atherosclerotic disease [3, 5], between 1996 and 2006 death rates from cardiovascular disease dropped 29.2% [8], probably due to a more well-educated patient population. Atherosclerosis is a systemic disease where fatty lipids deposit with inflammation, cells and fibrotic scar tissue on the vessel walls to form the initial basis for most cardiovascular pathology [5, 6, 8]. When blood flow is interrupted to the heart or brain, ischemia can cause a heart attack with infarct or a stroke with brain damage respectfully [6]. Lipids from the plaque core are originally naturally-occurring nonpolar organic hydrocarbons with many different molecular structures that are also insoluble in water to produce biologic functions for energy storage, protective membranes and many vitamins [2, 3, 5, 11]. Before clinical symptoms develop for cardiovascular disease as a heart attack or stroke, sub-clinical atherosclerosis progresses in large and small arteries leading into the heart and brain and also limbs and kidneys with calcifications that can now be imaged by computer tomography in the coronary artery [8]. The prevalence of such coronary calcifications in the United States for ages 32 to 39 years is now 5.5% and for ages 40 to 45 years 13.2% [8]. To better understand the problems, bulky atherosclerotic plaques that block normal blood flow are illustrated and further imaged in Fig. 1.

From basic Organic Chemistry and Inorganic Chemistry, free radicals are unstable reactive species containing an unpaired electron [1–3]. Free radicals in turn provide the fundamental incremental Chemistry unit to initiate reactive covalent structural-type pathology through basic unsaturated alkene C=C double-bond chain-growth crosslinking [1–4, 12]. In terms of chemistry nomenclature, an alkene is an unsaturated hydrocarbon with at least one C=C double bond and with less hydrogen atoms than a similar hydrocarbon alkane that is fully saturated with hydrogen atoms and no C=C double bonds and all single bonds [2, 3]. The odd electron radical is an electrophile and will readily pair with another electron to complete the valence and form a bond [1–3]. In one of the most common organic reaction mechanisms, radicals can achieve the octet valence shell through substitution commonly with halogenation chemistry by abstraction of an atom and one bonding electron that requires some energy to break a bond or alternatively through straightforward thermodynamics by simply directly adding to an unsaturated alkene C=C double-bond with no extra energy needed [2, 3]. In organic reactions, oxidation occurs by definition when there is a loss of electron density on a carbon atom [1–3]. Oxidation is subsequently generally defined as covalent electron-sharing bonding to a carbon atom with a more electronegative atom such as oxygen or breaking the less electronegative bond of hydrogen [2, 3]. Free radicals can initiate a thermoset reactive secondary sequence across C=C double bonds to create a solid polymer from a liquid-oil system. Free radicals attack C=C double bonds covalently through a chain reaction by propagating repetitive crosslink structure between each double bond in a common process termed chain-growth polymerization [1–4, 12–14]. Other selective terms for the reactive secondary sequence chain-growth polymerization are vinyl, olefin, addition or ethenic polymerization [1–4, 13, 14].

### 1.2 Lipid Peroxidation

As an alternate approach to understanding biological lipid crosslinking, an energy scheme to free-radicals related to the combustion of oils with oxygen and developed by Lavoiseir in 1789 has become a common chemical reaction design [1, 15, 16]. The proposed peroxidation of an average saturated tetrahedral alkane-type carbon backbone -CH_2_- group on a lipid hydrocarbon (LH) has then been presented by a basic chemistry with a carbon-hydrogen (carbon:hydrogen) bond dissociation and a free-radical initiator (R˙) to form an RH bond and lipid free-radical (L˙), modeled by Equation 1.

Equation 1 Initiation:R˙+LH→RH+L˙

**Fig. 2:**
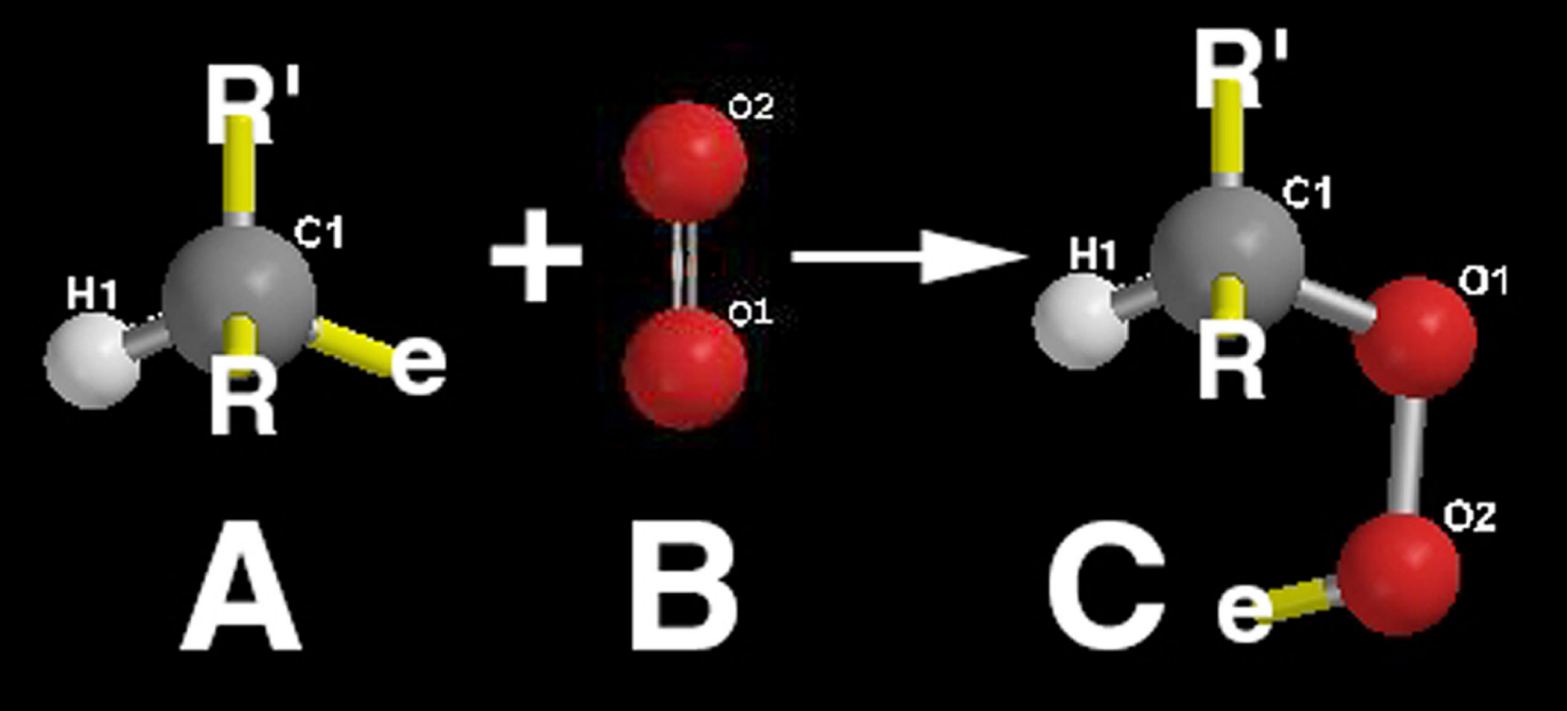
Lipid peroxidation of the carbon tetrahedral from (A) a saturated lipid -CH_2_- backbone group after hydrogen abstraction to form a carbon-centered free radical and then subsequent combining with (B) molecular oxygen to form a (C) lipid peroxyl free radical with large reactive electrophile swing rotation by two oxygen single bonds

Propagation has subsequently been expected to be produced from molecular oxygen if available then forming a peroxyl radical (LOO˙), as oxygen should readily accept an electron, Equation 2 and also Fig. 2 to better illustrate the peroxidation of a carbon-tetrahedral-centered free radical with large oxygen dihedral molecular swing through two separate single bond rotations.

Equation 2 $$\text{Propagation}:L˙+O_{2}\to LOO˙$$

The lipid peroxyl free radical in turn can form a hydroperoxide with a lipid peroxy:hydrogen bond if the necessary lipid carbon:hydrogen bond dissociation energy can be met once again also forming a new lipid free radical, Equation 3.

Equation 3 LOO˙+LH→L˙+LOOH

Loss of reactivity occurs by recombination of any two free-radical species, Equations 4–6.

Equation 4 Termination:2LOO˙→LOOOL or

Equation 5 2L˙→LL or

Equation 6 LOO˙+L˙→LOOL

Propagation steps during Equations 2 and 3 are barely appreciable for chain growth with only a peroxyl radical or hydroperoxide added and only by Equations 4 through 6 do chains actually really lengthen which are termination steps that end the reaction. As a result, chain growth through the proposed lipid carbon-tetrahedral -CH_2_- peroxidation reactions in Equations 1–6 appears minimal from a Chemistry standpoint with only two lipid chains combining, while energy for possibly two carbon:hydrogen bond dissociations proposed in Equations 1 and 3 are also necessary. But, for molecular space packing, termination can occur across three different molecular distances seen in Equations 4–6 to improve steric spatial arrangement possibilities for subsequent crosslinking between separate lipid chains. In addition, from a mechanomolecular perspective in terms of molecular movement through free-radical bond rotations shown by Fig. 2C and considered in Equations 4 and 6, the possibility for crosslinking by the aid of a molecule of oxygen and dihedral bonds having two complete rotations with some small length to access C=C double bond sites between two lipid chains should be appreciated. Further, because saturated lipids generally from animal fatty acids have been commonly identified as the major risk factor for atherosclerosis [2, 5, 6], alkane chemistry would appear to be a representative model.

However, when considering relevant biologic chemistry, certain inconsistencies exist within the current lipid-peroxidation model described through Equations 1–6. The basic saturated alkane-tetrahedral-type methylane -CH_2_- backbone lipid-peroxidation model proposed in 1981 instead was originally tested with a synthetic reactive conjugated aromatic-vinyl C=C unsaturated end-group styrene molecule and highly thermal active initiator with AIBN or azobisisobutyronitrile [15]. In addition, styrene was a chief reactive monomer from the Polymers Industry notoriously known to even polymerize by chain growth spontaneously without including free radicals [17]. In fact, use of styrene in the original study to provide Equations 1–6 rather than a lipid with a polyunsaturated fatty acid like linoleic acid was questioned [15]. Further, by Equation 3, the LOO˙ peroxyl radical formed during the propagation step reacts with the LH or -CH_2_- group of the lipid. The LH group is also known as an antioxidant which by definition replaces oxygen to minimize LOO˙ free–radical biologic damage, to then reform the L˙ radical. Subsequent polymerization chain-growth reaction does not then occur through Equation 3 due to minimal reactivity [18]. Chain-lengthen reactivity of the L˙ radical in the presence of LOOH is considered low enough so that inhibition is actually occurring where the cross-termination products (Equations 4–6) only occur as shown [18]. The R˙ + LH reaction sequence with O_2_ (Equations 1–6) to LL or possibly LOOOOL and LOOL has in fact been termed a Free-Radical Inhibition by Transfer Mechanism in Polymer Science [18]. Regardless of early confusion with the alkane lipid model, current alkene chemistry is starting to overtake lipid alkane chemistry with more thermodynamic favorable hydrogen dissociation particularly catalyzed with enzymes and most identified with a bisallyic double bond (R-C=C-C=C-R) to produce the initial lipid free radical in the model developed through Equations 1–6 [16, 19]. In addition, although hydroperoxides have also been considered as the primary products for unsaturated lipid peroxidation by Equation 3, cleavage of hydroperoxides is now thought to continue by decomposition to more stable aldehydes and ketones where chain lengthening is not measured [20].

### 1.3 Polymer Science Oxidation

Alternatively, from a Polymer Science perspective, through molecular oxygen chemistry, vinyl C=C end-group systems have shown related cross-linked surface oxidation in rubber and silicon [21–24]. In fact, oxygen at high concentrations will diffuse and pack at molecular size to increase the top surface layer crosslink density of C=C vinyl-end group systems [21–25]. Resultant surface crosslink oxidation subsequently reduces oxygen diffusion into the bulk material [21–24]. Still as a general rule, molecular oxygen will also complex with free radicals to form more stable peroxy radicals, reduce crosslinking and is considered an inhibitor of free-radical chain-growth polymerization for thermoset resins [4, 18, 25, 26]. For practical thermoset resin application, free-radical polymerization can proceed in thicker thin films in the deeper layers yet molecular oxygen can limit polymerization at the surface interface leaving a tacky top layer [27]. Nonetheless, oxygen is not considered a good inhibitor of free radicals in a closed resin container because of the limited amounts [18]. Since oxidation takes place as an event that reduces carbon electron density [1–3], oxidation can also be defined by a highly electronegative free radical taking a pi (π) electron from a C=C double bond. When free radical concentrations are high in the presence of C=C double bonds in addition to oxygen, loosely held π orbital electrons should then be considered for Chemistry interaction especially for transitions occurring with liquid alkene oils into solid polymer products [1–4, 12–14].

### 1.4 Thermodynamics of Hydrogen Abstraction and Comparisons for Single Bond Dissociations

The hydrogen abstraction mechanism proposed in Equations 1 and 3 to produce a free radical in fact requires considerably more energy to dissociate a carbon:hydrogen single bond compared to breaking a peroxy oxygen-oxygen (oxygen:oxygen) single bond for hydrogen peroxide. Dissociation of a carbon:hydrogen single bond even requires more energy than an equally substituted carbon-carbon (carbon:carbon) single bond, Table 1 on Bond Dissociation Energies for Homolytic Cleavages A:B → A∙ + ∙B [2, 28]. Further note that when organoperoxides form, RO:OR single bond dissociation energies are even substantially lower than for peroxide or HO:OH so that organic peroxy substitutions will lower the reaction rates for free-radical generation [2, 28]. Also, as a basic comparison the standard biologic energy measure of hydrogen phosphate produced from adenosine triphosphate and water is over an order of magnitude lower than any carbon:hydrogen single bond dissociation [2, 28, 29]. Because lipid peroxidation, defined by a peroxide bond forming at a -CH_2_- carbon backbone tetrahedral position can also really be a form of oxygen inhibition of free radicals [18], then the fundamental Chemistry reaction must be practically reexamined according to basic Chemistry and Polymer Science. When unsaturated lipid alkene crosslinking by reactive secondary sequence across C=C double bonds is understood at a more advanced level, the subsequent biocomplexities with peroxyl radical recombination during termination can then be better explored more thoroughly for Medical conditions.

### 1.5 Fenton Reaction

Transition metals have been widely known to act as cation redox catalysts [1, 4, 30]. By the Fenton reaction, Equation 7, an electron is transferred from a metal species to promote breaking of the peroxide bond (RO:OR) at physiologic temperature [30, 31]. Biologic peroxides would include hydrogen peroxide and other organoperoxides [4, 5, 29–31] to increase the possibility of providing free radicals with an unpaired electron.

Equation 7 $$RO:OR(\text{initiator})+M^{++}(\text{Redox promoter})\to RO{:}^{-}+˙OR+M^{+++}$$

RO:OR is an oxidizing agent that accepts electrons and the M^++^ is a reducing agent that donates electrons so together both are termed a redox couple in the formation of the ˙OR free radical initiator. Similar redox initiator/promoter systems with a metal salt for inducing the decomposition of a peroxide to make free radicals are used in the fabrication of thermoset polymer parts [4, 31].

### 1.6 Reactive Secondary Sequence Chain-Growth Thermoset Polymer Model

Reactive secondary sequence thermoset-polymer chain-growth lengthening is presented through basic Chemistry and Polymer Science [1–4, 13, 14]. Basic resins for industrial application that form thermoset polymers include the most common as polyesters, vinyl esters and epoxies which account for about 90% of all polymer matrix composites [14, 32]. For classification, two major forms of polymers are termed thermoplastics or thermosets [3, 4, 13, 14, 33]. Thermoplastics contain linear or slightly branched chains that entangle lightly so that heating or pressure allows chains to slide past one another and the solid polymer can then remelt without chemical modification on heating [3, 4, 13, 14]. Thermosets on the other hand crosslink by forming covalent bonds into a network of chains to an irreversible state of hardness so that heating does not cause polymer softening below decomposition temperatures [3, 4, 13, 14]. Free-radical chain-growth polymers are included as thermosets by virtue of the vast exothermic crosslinking between the molecules [4] during a cure process where a low viscosity liquid converts to a hard solid [3, 13, 14]. During thermoset polymerization reactions, as crosslinking increases viscosity increases where soluble polymers form in the sol stage so that branched clusters develop eventually to infinite size into the gel stage and continue to crosslink basically to one large covalent bonded molecule called a polymer network [12, 14, 35, 34]. As the crosslink density of the thermoset network increases, the modulus (approximately stiffness) also increases [4, 13, 14, 27, 34–37]. Further, from a biological perspective synthetic epoxidized thermoset resins produced from soybean oil have demonstrated that modulus further increases by adding ring structures to the linear polymer chain [38–42]. Regarding a biological thermoset polymer model, resins that represent the alkene chemistry with C=C double bond functional reactive groups include polyester and vinyl ester. Also, isoprene-like rubbers have similarities for the thermoset C=C double bond reactive secondary sequence chain-growth addition polymerization from a liquid or oil to a solid.

**Fig. 3:**
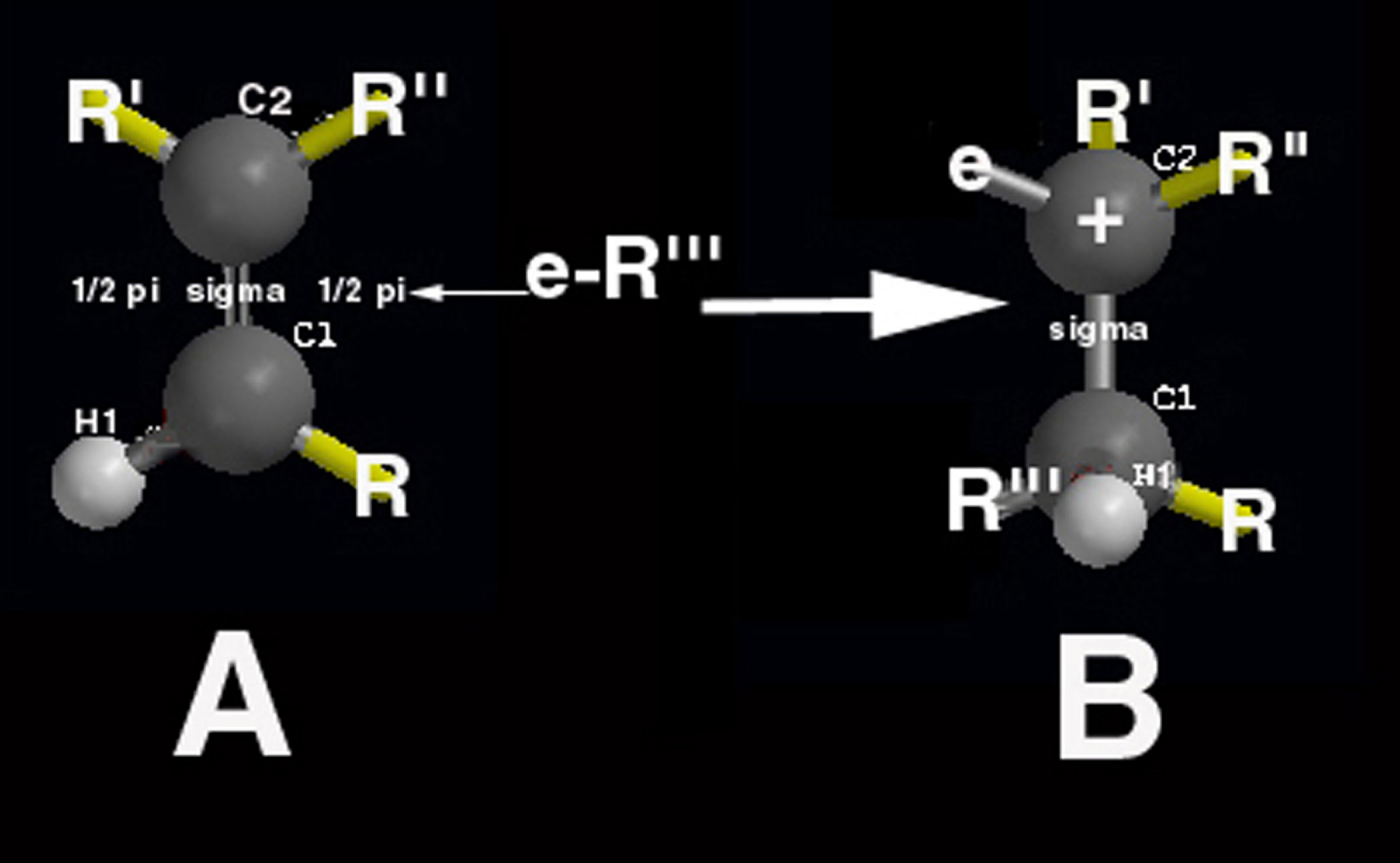
Reactive secondary sequence where (A) a lipid planar C=C alkene π bond is attacked by a free-radical electrophile with unpaired electron, e-R”’, to (B) form a carbon tetrahedral for an R”’ σ single bond and produce a tertiary carbon radical containing a new unpaired electron on the opposite carbon atom ready to react through another C=C alkene π bond

One important characteristic of the C=C double bond is the planar non-rotating nature that further provides ease of accessibility to the outer π orbital for other reactants while sigma (σ) single bonds on the other hand rotate [1–3, 26]. The most obvious free-radical C=C events pertain to addition reactions, Figs. 3A–B, that can also include propagation through repetitive reactive secondary sequence thermoset chain-growth polymerizing into large macromolecules for significant chain-lengthening events [1–4, 13, 14, 18, 26]. Following covalent bonding at C-1 between the free radical and the C=C π bond electron in Fig. 3A, the new carbon radical on the opposite carbon atom at C-2 in Fig. 3B is in transition between a planar form that should closely resemble the tetrahedral structure above. The new unpaired electron on the carbon radical from the original disrupted π bond is highly reactive and can then produce a new reactive secondary sequence with another alkene C=C π bond. Propagation by extensive reactive secondary sequence chain reactions through C=C double bonds of a lower molecular weight liquid one molecule at a time can then produce large stabilized solid thermoset macromolecules through conversion of π bonds to σ bonds [3, 4, 13, 14]. Lipid peroxidation by molecular oxygen can also occur with the lipid carbon free radical to inhibit chain growth through other C=C π bonds or also crosslink by recombination as a termination step. Regarding a possible atherosclerosis thermoset polymer model, free radicals attack C=C double bonds in polyunsaturated fatty acids that increase the risks for cardiovascular disease, cancer and many other medical conditions [43].

Of further note, polymer diffusion is approximately inversely proportional to the square of the molecular weight [4, 44], so that shorter chains diffuse more rapidly to bring reactants closer together. Also, longer polymer chains of higher molecular weight entangle to reduce polymer movement thus increasing viscosity whereas shorter chains can more easily disentangle [1, 4, 44]. In addition, alkanes generally form solid fats by entanglement whereas planar C=C double bonds reduce entanglement to form oils [2, 3, 5, 11, 44]. Of more importance toward a biologic thermoset model, H_2_C=CH-R vinyl end groups have greater reactivity than vinyl side-chain groups due to steric spatial arrangements [45]. Vinyl C=C end groups in fact are preferred over internal C=C groups on the carbon chain backbone for crosslinking unsaturated polyester and vinyl ester polymer systems so that the C=C function is easily accessible [26, 46, 47]. Conversely, internal C=C bonds in conjugated π systems compared to end group C=C bonds are more associated with antioxidants and delocalization of free radicals although reactive secondary sequence lipid chain-growth addition is still possible [2, 3, 48]. In terms of a possibly high potential for biologic available vinyl groups, polyisoprenoids are long-chain highly polyunsaturated polymers of the repeating 5-carbon monomer isoprene (2-methyl-1,3-butadiene) with two vinyl groups that exist in the plasma cell membrane [2, 29]. Further, polymer chain-growth reactions are some of the most fundamental free-radical Organic Chemistry C=C reactions that can occur with alkene unsaturated C=C double bonds [2, 3].

### 1.7 Organic Chemistry Carbon-Carbon Double-Bond Oxidation to Aldehydes and Ketones

**Fig. 4:**
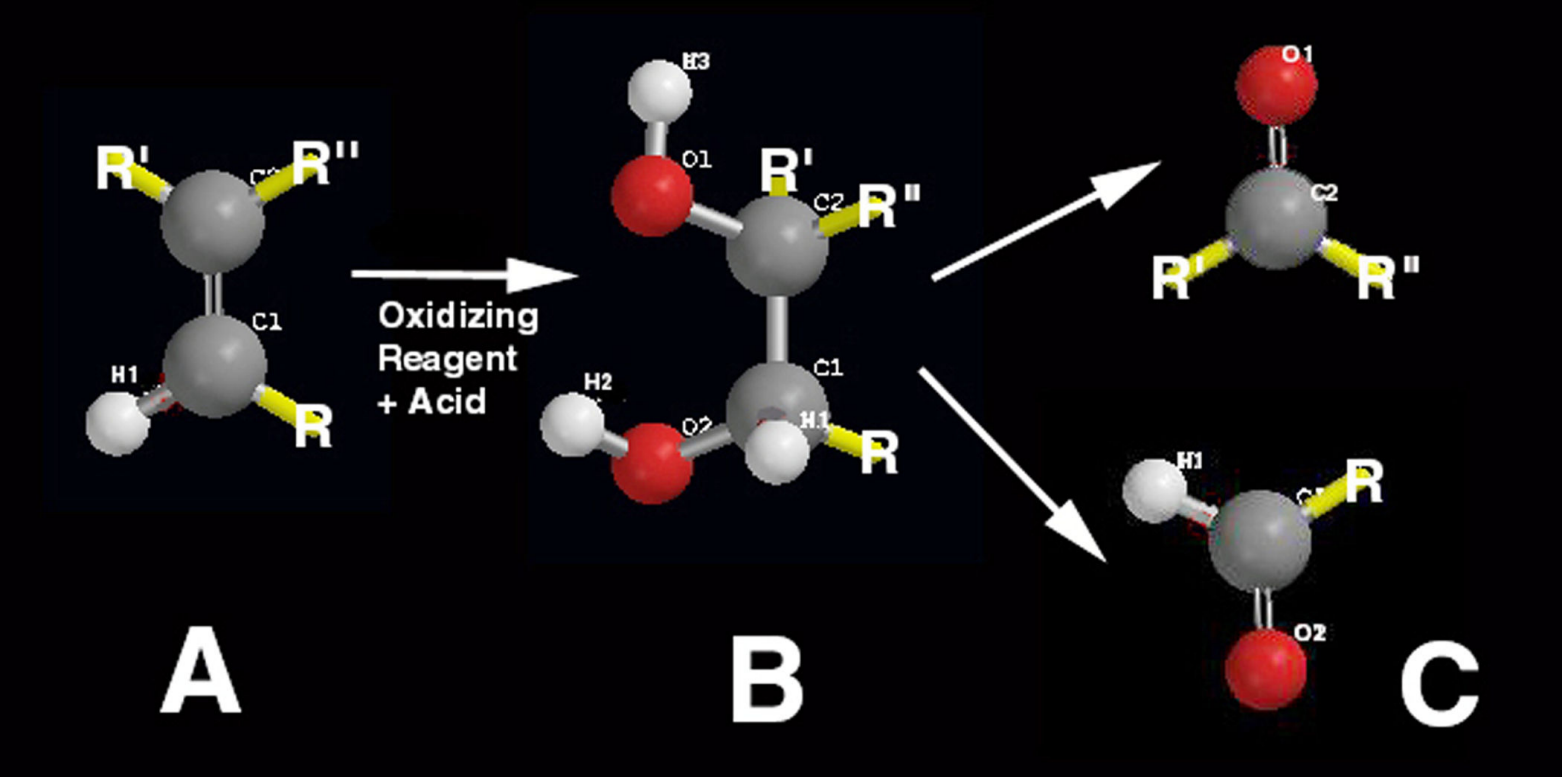
(A) Lipid Alkene (B) Glycol Oxidation intermediate (C) Oxidative Cleavage into Ketone top and Aldehyde bottom during highly concentrated free radical or combined acidic conditions

Also, other common potential alkene products such as aldehydes and ketones by oxidative cleavage of polyunsaturated lipid C=C bonds [2, 3] should be acknowledged to better understand the reactivity and crosslinking potential of smaller chain-length lower molecular weight alkenes. If oxidant species are concentrated or in an acidic solution, diol hydroxylation can occur across the reactive C=C alkene with subsequent breakdown into aldehydes or ketones [2, 3], Fig. 4. Recent work on hydroperoxides formed through Equation 3 on lipid peroxidation confirms unsaturated lipid common decomposition to more stable aldehydes and ketones [20]. For lipids, the aldehyde would be the common product rather than the ketone due to the R-CH=CH-R reactive nature of the linear chains. By shortening the lipid into smaller aldehyde molecules, Fig. 4C, that can move easier into tight spaces between lipid chains thus reducing spatial steric hindrance, the possibility of a free-radical C=C double-bond crosslinking longer chains should greatly increase. In terms of reactivity, smaller molecules increase diffusion at an exponential rate [4, 44], whereas longer polymer chains of higher molecular weight entangle to reduce polymer movement [1, 4, 44]. Most importantly, by breaking lipids down, possible H_2_C=CH-R vinyl end groups may form for increased polymerization reactivity. Further, biologic catalytic proteins called enzymes can bring all reactants with acids buffered and radicals delocalized to provide energetic solutions to overcome difficult bond dissociation chemistries [1, 5, 11, 29].

### 1.8 Mitochondria Energy Synthesis and Biocomplex Reactions

In terms of Biology interactions, cell mitochondria energy synthesis is fundamental to the multiple varied types of biocomplex reactions. During mitochondrial energy production electrons are produced through the electron transport chain and acids develop from the proton gradient [5, 11, 29, 49]. Consequently, as an important fundamental physiologic understanding, when oxygen is not available to form water during any pathology or biologic condition, hypoxic cellular respiration by mitochondria will produce both electrons and also acid such as hydronium or the hydrated hydrogen ion (H_3_O^+^) [5, 11, 29, 49]. A build-up of both free-radical electrons and acids would possibly then meet both alkene C=C common conditions for either reactive secondary sequence with repeated chain growth or oxidative cleavage lipid breakdown to an aldehyde [2, 3]. Therefore, with low oxygen more biocomplex reactions would subsequently ensue when considering both of the events for increased free radicals and lower pH. In fact, complications from atherosclerotic ischemic diminished blood flow induce mitochondria to produce excessive free radicals from the electron transport that further promote atherosclerosis [9, 50]. Therefore, the mitochondria have been considered as a possible important site for drug therapy to treat most diseases related to reactive oxygen species [49, 51]. Pharmaceutical design includes consideration for smaller low molecular weight molecules that diffuse adequately to the mitochondria at low toxicities for long-term administration and higher accumulation [51].

During free-radical lipid chain lengthening, several common fatty acids are potentially capable of entering into a reactive secondary sequence C=C process. Polyunsaturated fatty acids with C=C double bonds are not only located in the plasma cell membrane, but also on circulating plasma triglycerides [3, 5, 11, 29]. For example, the common lipids arachidonic, linoleic and oleic contain carbon atoms and C=C double bonds in the ratios of C20:4, C18:2 and C18:1 respectively [1–3, 5, 28, 29]. As previously mentioned, polyisoprenoids are long-chain highly polyunsaturated polymers of divinyl 5-carbon isoprene (2-methyl-1, 3-butadiene) that also exist in the plasma cell membrane [2, 3, 29]. Oxidative cleavage of alkenes not only breaks down lipids through hydroxylation [2, 3], but with additional lipid C=C double bonds can potentially further produce shorter-chain more reactive vinyl end-group alkene C=C aldehydes. Shorter hydrocarbon breakdown products reduce molecular entanglement with improved diffusion [1, 4, 44] and with C=C vinyl end groups then improve the possibilities of crosslinking the long-chain alkenes by reactive secondary sequence chain lengthening [26, 45–47]. In fact, the short-chain reactive vinyl C=C end-group aldehyde, acrolein *(CH_2_ = CH – CHO)*, and other short chain aldehydes identified with lipid peroxidation have been associated with numerous free-radical pathologies such as cancer, atherosclerosis and diabetes [52–56].

Difficulty in providing a well understood cellular mechanistic view of lipid free-radical chemistries is subsequently also due to complex events that result from the initiating free-radical intermediates, which are not easy to isolate, especially in a diverse pathological bioenvironment. In addition, free-radical chain-growth polymers become increasingly structural with greater molecular weight [1–4], and less able to dissolve in blood-plasma fluids for analysis [2, 3]. Failure to identify the initial reactive secondary sequence free-radical products that form across the C=C double bond then makes a cellular mechanistic view that much harder to predict. Fortunately, with regard to diverse downstream biocomplexities in coronary heart disease, researchers are now starting to consider multiple initiating roles and healthy modifiers concerning free-radical pathogenesis models that identify oxidized lipids, serum lipoprotein fractions as low density lipids (LDLs) or high density lipids (HDLs), animal products like red meat or egg yolk, saturated fatty acids, unsaturated fatty acids, inflammation, and infections [3, 5, 11, 57–59]. In terms of providing important new chemistry for reactive secondary sequence polymer chain growth in atherosclerosis, even the different types of unsaturated fatty acids for C=C double bond numbers, positions and cis/trans stereoisomer chemistry are being investigated for a relationship with coronary heart disease [3, 6, 11, 59]. Also, in terms of Regenerative Medicine with approaches for treatment from Polymer Science, new free-radical inhibitors that practically stop reactive secondary sequence chain-growth polymerization during an induction phase and retarders which merely slow the chain-lengthening process down [4, 18 26] can further be tested to control pathology.

### 1.9 Antioxidant Tests

A current problem exists on administering supplemental nutrients to prevent cardiovascular disease. Although, reactive free radical species are considered the chief source for advancing the complications of atherosclerosis and cardiovascular disease [10, 60, 61], large-scale cardiovascular clinical trials using antioxidants such as vitamin E, vitamin C and beta-carotene have not proven to be effective [10, 59–66]. Reactive oxygen species are also thought to promote cancer [49], but similar clinical trials for cancer suggest that vitamin supplements are not effective in reducing risks during treatment [67]. In fact, several investigators have assumed beyond the great biocomplex events in pathophysiology that complicate healing, the commonly supported vitamin E may not be stable enough to be an antioxidant but rather provides other types of benefits [68–73]. To better understand discrepancies between measured vitamin antioxidant potentials and clinical failure perhaps the antioxidant tests should be considered. Current antioxidant tests that created the indices comparing different vitamins with activities of other antioxidants are primarily based on changes between the oxidation of a sample and free-radicals from a stable source, metal ions through a Fenton reaction or a physiologic-relevant reactive species such as the hydroxyl radical [74–77]. The original antioxidant testing by Burton and Ingold in 1981 used thermal changes as an indication for free-radical complexing with a sample [15]. The most current common antioxidant tests use colorimetric assays with uv-vis spectroscopy that examines optical adsorption at a characteristic maximum peak in the visible region of the electromagnetic spectrum between about 400–700 nm [74–77]. In the assay sample-free-radical complex, adsorption occurs at a maximum peak that can be measured from a control standard. When the antioxidants are added, the free radicals are sequestered and the measured assay peak is reduced depending on the vitamin concentration. Many different colorimetric antioxidant assays are used today to include thiobarbituric acid reactive species (TBARS), total antioxidant capacity (TAC), total antioxidant potential (TAP), total radical adsorption potential (TRAP), Trolox equivalent antioxidant capacity (TEAC), curic ion reducing antioxidant capacity (CUPRAC) and ferric reducing antioxidant power (FRAP) [74–77]. High performance liquid chromatography (HPLC) has further been used for a colorimetric antioxidant assay with a fluorescence method [77]. However, many problems may occur to interfere with the accuracy of antioxidant indices. For example, thermal changes indicating covalent bonding was not previously considered in the original lipid peroxidation model [15]. The use of different solvents [75, 77, 78] could tie up free radicals by competing with the antioxidant and also influence light adsorption in different manners. Further, thermal incubation periods up to 50 degree C [74, 75] could denature protein and enzyme antioxidants used as other important measures within the indices. In terms of the importance for atherosclerosis regarding thermal denaturing problems, HDLs contain proteins and enzymes that are thought to exert antioxidant and anti-inflammatory effects by inhibiting LDLs considered one of the main initiating factors for atherosclerosis [79]. Because the colorimeter measurements are based on adsorption properties of conjugated bonds [2, 3], differences in conjugated ring structures and conjugated linear structures might be considered. Also, the calibration standards may need uniform standardization through committees. Antioxidant colorimetric tests are based entirely on colored changes with adsorption in the visible-light electromagnetic spectrum. Conversely, free-radical covalent bond structural formation of a solid polymerized product from a liquid oil involved in vascular atherosclerosis obstruction is hypothesized to occur by a different chemistry through a reactive secondary sequence of an alkene C=C double bond for the current testing.

## 2. EXPERIMENTAL DETAILS

### 2.1 Fenton Metal Polymerization Model

**Fig. 5:**
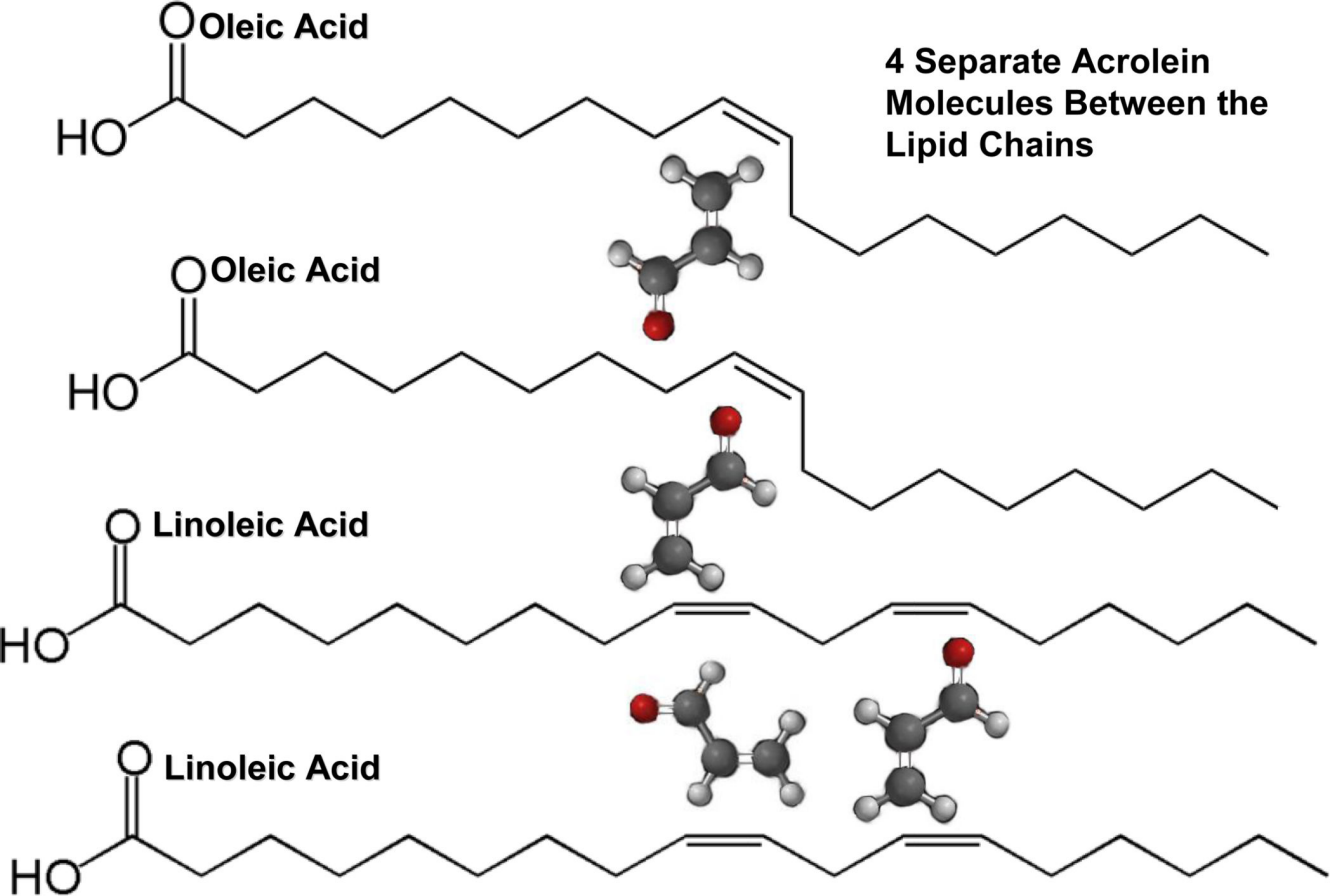
Steric lipid reactant C=C double-bond spatial possibilities in one-plane relationships for crosslinking the fatty acids oleic acid and linoleic acid with acrolein using a free-radical generating redox couple consisting of 4wt% each with cobalt naphthenate and dibenzoyl peroxide. Three-space crossover interactions bring C=C bonds closer together to provide greater reactivity

In order to determine the possibility of reduction metal Fenton-type reactions occurring, a 90:10 oleic:linoleic lipid oil/acrolein model, Fig. 5, was used with cobalt naphthenate and dibenzoyl peroxide, all Sigma Aldrich, St. Louis, MO, with undetermined mixing conditions. The cobalt naphthenate is a common organo-metallic naphthenic acid reducing agent for accelerating peroxide RO:OR bond dissociation to produce free radicals with 6% cobalt [80]. In essence, cobalt naphthenate represents a cobalt atom sequestered inside four small saturated ring molecules each with a conjugate carboxylic acid base for a side group [80] that provides a diffusion medium for the Fenton metal atom and subsequent compatibilization with other organic reactive hydrocarbons [80]. Initial concentration used a 50:50 mixture of lipid oil and acrolein with 4wt% of dibenzoyl peroxide and 4wt% cobalt naphthenate (0.24wt% cobalt).To better understand crosslinking potential with the vinyl acrolein end-group and the lipid C=C double bonds, the lipid oil concentration was increased up to 100% and the acrolein was reduced to 0.0% with the same redox cure agent concentrations at 4wt%. Further, to better appreciate the Fenton metal redox interaction with the benzoyl peroxide, the 50:50 lipid:acrolein mixture was combined with only the dibenzoyl peroxide and no organo-metallic cobalt naphthenate.

### 2.2 Chain-Growth Polymerization of Vitamin A and Beta Carotene

**Fig. 6:**
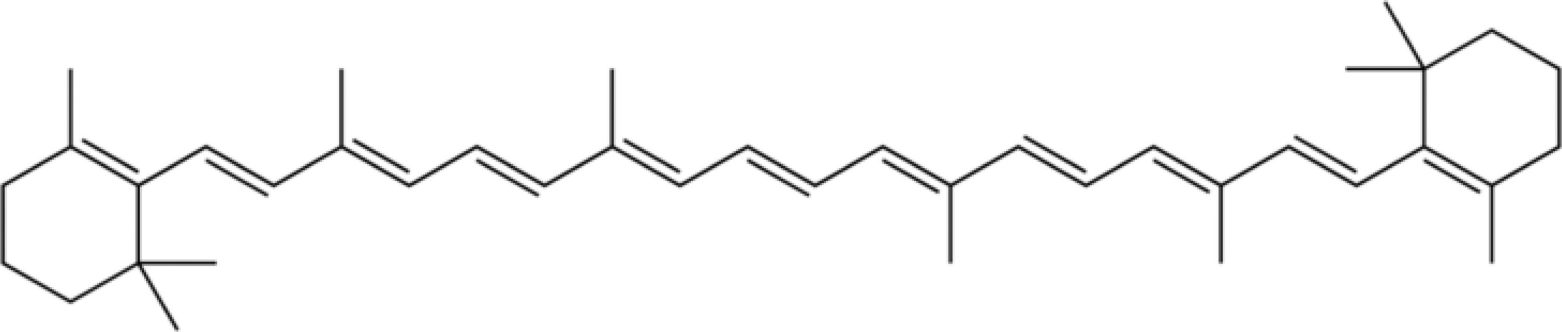
β,β-carotene conjugated C=C double-bond π system produces a deeply colored pigment and can be cleaved enzymatically into two molecules of vitamin A [2, 3]

Vitamin A and β,β-carotene are carotenoids that act as antioxidants and contain multiple conjugated isoprene units [48] with potential to react by chain-growth polymerization through the C=C double bonds. In fact, addition reactions can occur with the highly conjugated carotenoids where free radicals most often delocalize toward the first terminal C=C double bonds (C-7,8) on the linear chain immediately next to the rings on either end [48]. The molecular structure of the extensive C=C conjugated double bonds is shown in Fig. 6.

To test the potential for free-radical activity through antioxidant C=C double bonds, 100 capsules of vitamin A 8000 IU/capsule and β,β-carotene 25,000 IU vitamin A/capsule, both Nature Made, Mission Hills, CA, were incised and squeezed into separate containment wells. Both vitamin A and β,β-Carotene were supplied with low viscosity unsaturated oils from fish liver/soybean and corn/soybean respectively in non-bovine gelatin capsules with glycerin and water. In regards to the antioxidant oil-base viscosities, unsaturated vegetable or fish oils have a high proportion of planar C=C double bonds that lower the melting points by removing a bond rotation when compared to solid animal fats that entangle extensively more through the saturated single bonds [2, 3]. For example corn oil is predominantly an unsaturated oil as 35% oleic C18:1 and 45% linoleic C18:2 [2]. Each antioxidant and respective oil base combination was then incorporated with increasing concentrations of Fenton-metal cobalt naphthenate reducing agent and dibenzoyl peroxide oxidizing agent over 3 days, both Sigma Aldrich, St. Louis, MO, to test the possibility of reactive secondary sequence chain-growth polymerization by observing viscosity changes from the original low viscosity liquids.

### 2.3 Free-Radical Polymerization Cure Shrinkage Method

As covalent σ bonds form by replacing C=C double π bonds during reactive secondary sequence chain growth, polymer chains draw together from more distant van der Waals intermolecular attraction forces and chain-entanglement equilibrium distances to closer covalent distances with increasing chain entanglement that reduce bulk volume by linear/volumetric cure shrinkage [13, 14, 26, 81]. Double-bond conversion to single bonds also forms an exothermic polymerization of about 10–20 kcal/mol even without extra energy added at room temperature [4]. Consequently, solutions of unsaturated lipids that undergo thermoset free-radical chain growth will also produce linear/volumetric cure shrinkage without added energy. In order to show the basic unsaturated lipid free-radical cure shrinkage, a similar solution from the previous testing using a 90:10 oleic:linoleic lipid oil/acrolein model with the Fenton-metal free-radical agents as benzoyl peroxide and cobalt naphthenate were combined in clear styrene cylindrical vials to visually observe and measure the thermoset polymerization shrinkage. When 71% lipid oil was combined with 28% acrolein and 0.5% benzoyl peroxide and 0.5% cobalt naphthenate in 5 separate clear plastic vials with an inside diameter of 20.0 mm, shrinkage could be measured by the following formula in Equation 8:

Equation 8 %Shrinkage=100×(original volume−volume after shrinkage)/original volume

### 2.4 Antioxidant Cure Shrinkage Testing with Free-Radical Inhibitors and Vitamin E

**Fig. 7:**
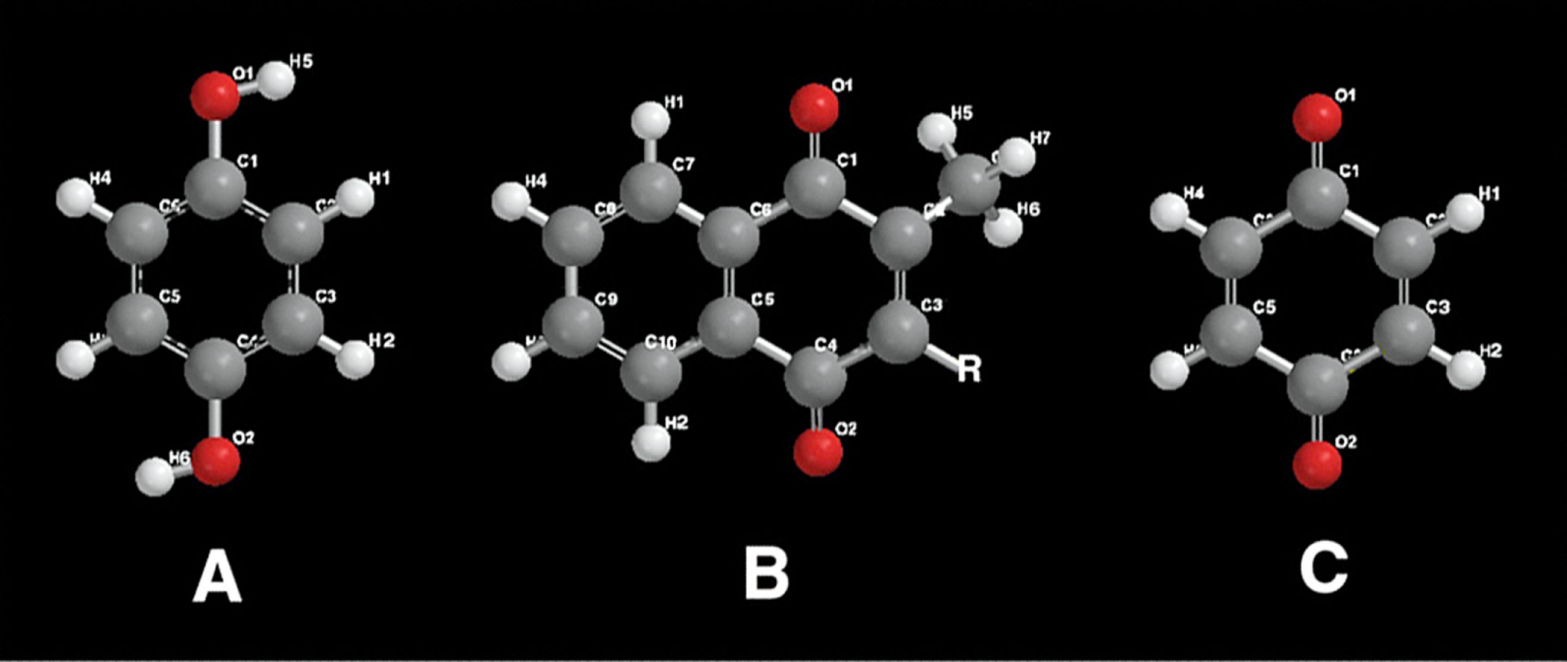
(A) Hydroquinone (B) Vitamin K shown in the reduced quinone form (C) Quinone

Hydroquinone, Fig. 7A, as a conjugated planar ring molecule in the form of an aromatic ring with two hydroxyl groups in a para position is perhaps the most common free-radical inhibitor from the Polymer Industry. Structurally, hydroquinone appears similar to the quinone vitamin K, Fig. 7B [2, 4, 82], but potentially more diffusively active due to a much smaller molecular size. In addition, vitamin K has potential toxicity that is resolved by reduction of the quinone form to a hydroquinone as the safe active species [82]. Vitamin K further includes at least one C=C double bond on the R group [82]. Hydroquinone has two oxygen dihedral bonds able to rotate with permanently exposed dipoles so that as a nonpolar entity also has capable polar water interaction to be carried through the blood stream, diffuse through biological fluids and then be excreted. In fact, the hydroquinone form of vitamin K with two hydroxyl groups has potential polar water solubility and shows enhanced delivery through biological fluids *in vivo* [82]. Conversely, the nonpolar quinone vitamin K is practically insoluble in aqueous solution [82]. Quinone, Fig. 7C, is the oxidized form of hydroquinone and will reduce back to hydroquinone [2].

**Fig. 8:**
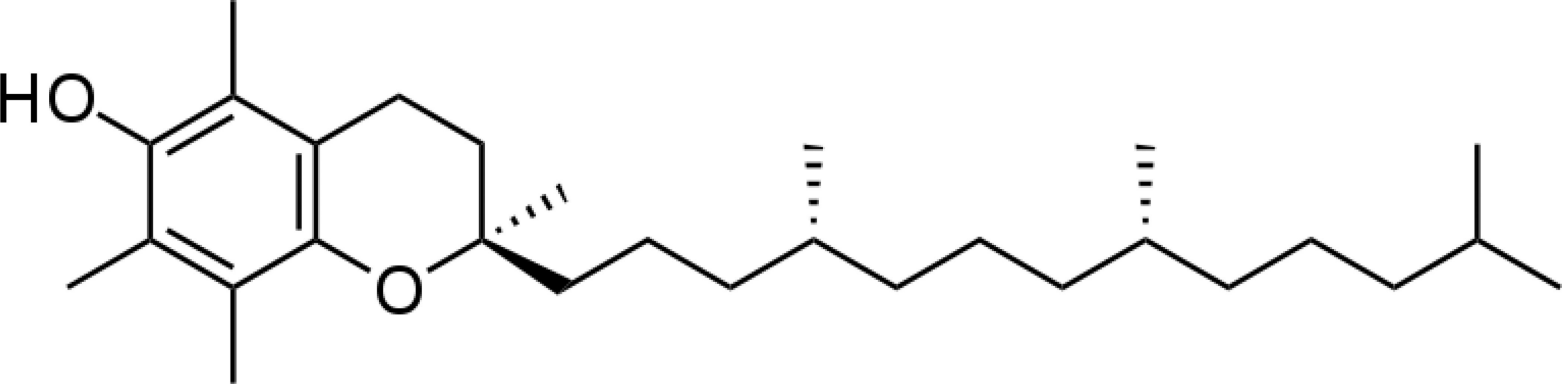
Vitamin E molecular structure [3]

Vitamin E as the α-tocopherol form for antioxidant comparison, Fig. 8, has some similarity to hydroquinone to possibly delocalize free radicals into a phenyl ring from an ether cyclic ring. However, α-tocopherol is a much larger hydrophobic or nonpolar molecule than hydroquinone and from laboratory observations is practically insoluble in water whereas hydroquinone will dissolve easily to diffuse through water. From basic Organic Chemistry, in terms of subsequent molecular affinity charged free radicals would tend to associate more towards polar biologic fluids [2, 3]. By similar Organic Chemistry, free radicals should be expected to combine more with the water soluble polar hydroquinone than nonpolar cell membranes and the vitamin E shown as α-tocopherol.

To compare the antioxidant behavior between vitamin E and the common Polymer Science free-radical inhibitor hydroquinone, lipid:acrolein models at 46:46 weight percents were mixed with Fenton benzoyl peroxide and cobalt naphthenate redox couples at 4wt% each for generating free radicals. For each group, 5 clear cylinder styrene vials were filled with the lipid:acrolein solutions containing the free-radical generating cobalt naphthenate/benzoyl peroxide redox couples as a baseline control and further similar groups were combined with different amounts of either vitamin E ((±)-α-tocopherol) or hydroquinone (ReagentPlus 99%) as shown by Table 2 (both Sigma Aldrich, St. Louis, MO). Shrinkage was thus calculated over time for 50 hours by measuring the differences between the original level for the volume and volumetric shrinkage polymerization level with Equation 8.

### 2.5 Statistical Analysis

The level for significant statistical differences was set at α = .05. The Coefficient of Determination (R^2^) was further used to provide analysis as the percent of the total variation in the values for Y that could be explained by the regression where 1.00 = 100%.

## 3. RESULTS AND DISCUSSION

### 3.1 50:50 Lipid Oil: Acrolein Mixture with a Fenton Metal/Peroxide Free-Radical Reaction

**Fig. 9:**
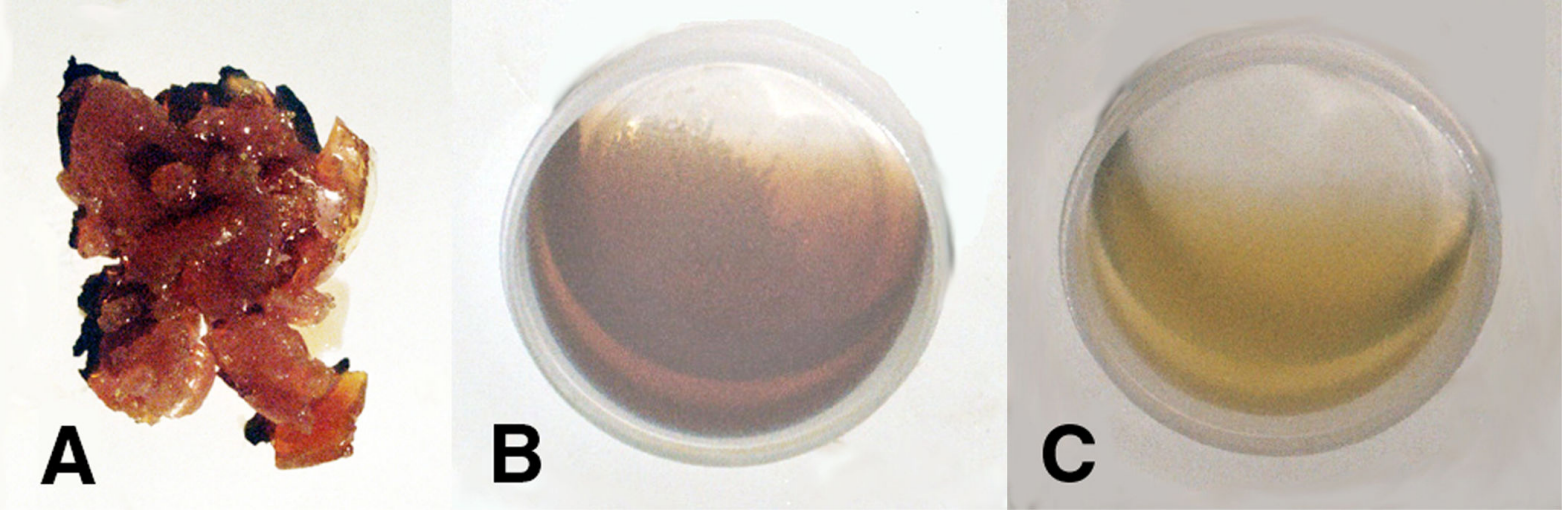
A–B. (A) Rubbery-type adhesive solid gel produced by lipid chain lengthening seen with mass removed from the plastic polyethylene reaction container after three months at a lipid:acrolein 50:50 ratio wt% concentration and free-radical redox couple of 4wt% each for dibenzoyl peroxide and cobalt naphthenate. (B) Acrolein removed for 100% lipids with a free-radical redox couple of 4wt% each for dibenzoyl peroxide and cobalt naphthenate. Since cobalt naphthenate is an intense deep purple color, the clear lipid oils with acrolein tint to a brown in 9B. (C) Reducing agent cobalt naphthenate removed for lipid:acrolein 50:50 ratio wt% concentration and free-radical oxidizing agent dibenzoyl peroxide of 4wt%

Initial concentration using a 50:50 mixture of lipid oil and acrolein with 4wt% cobalt naphthenate (0.24wt% cobalt) and 4wt% dibenzoyl peroxide produced a reactive secondary sequence solid rubbery-type sticky gel within about 4 days, Fig. 9A photo after three months following mass measurement. Over the 3 month period, by increasing the lipid oil and reducing the acrolein with the same redox cure agent concentrations at 4wt% each, the solidified gel stage took progressively longer or attained only a gluey state such that mixture viscosity decreased with less acrolein. Moreover, after three months no thermoset crosslink gel chain lengthening could even be detected at any appreciable level with 100% lipid oil and no acrolein, Fig. 9B. Also, when no acrolein was added much of the dibenzoyl peroxide particles did not dissolve and remained at the bottom of the reaction container, Fig. 9B. Viscosity increases related to acrolein concentration were highly dependent at some yet uncertain exponential rate. For comparison, a 50:50 mixture of lipid oil and acrolein with 4wt% dibenzoyl peroxide and no cobalt naphthenate is shown in Fig. 9C. When the 50:50 lipid:acrolein mixture was combined with only the dibenzoyl peroxide and no organo-metallic cobalt naphthenate, chain lengthening following three months duration was again almost absent, Fig. 9C. The lower molecular weight acrolein evaporates so without the cobalt naphthenate reducing agent to promote peroxide bond dissociation and then minimal lipid crosslinking, a certain amount of the reactive α,β-unsaturated aldehyde has evaporated in Fig. 9C. In all, both the acrolein short-chain cross-linking agent and organo-metallic cobalt Fenton-type reducing agent were both fundamental in order to produce thermoset reactive secondary sequence lipid chain lengthening into a gel as a solid crosslinked mass. In addition, over an extended approximate period of one year for both Figs. 9B and 9C, lipid viscosity increased and contained diffuse islands of gelation associated with the dissolving white benzoyl peroxide particles. Also, over time the rubbery solid mass in Fig. 9A continued to crosslink for increased structure by hardening and stiffening.

**Fig. 10:**
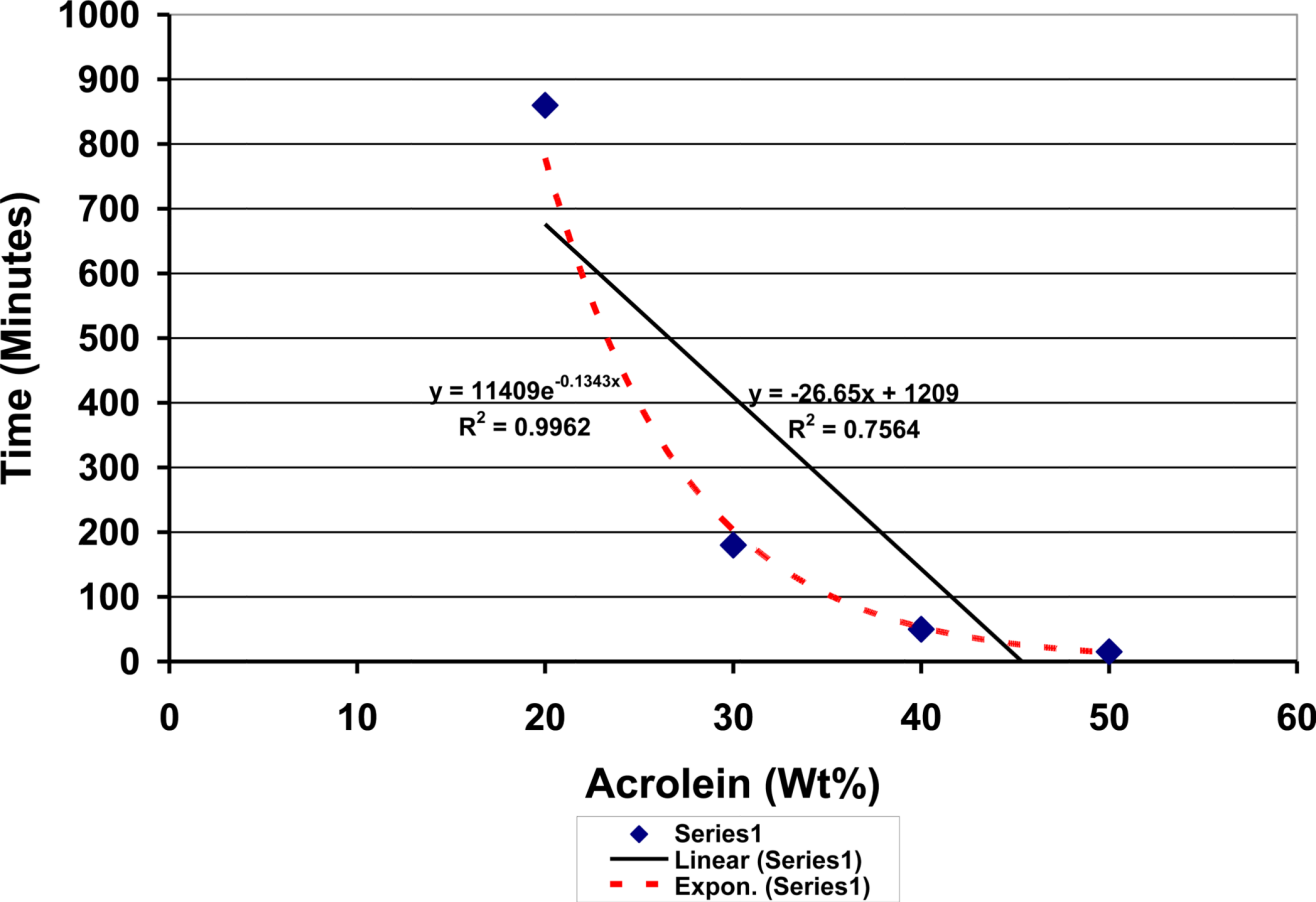
Chart depicting decreasing time with increasing acrolein concentrations for the complete disappearance of the 4wt% dibenzoyl peroxide oxidizing agent with 4wt% cobalt naphthenate reducing agent in a lipid oil solution over about one day.

The rate of disappearance for the 4wt% dibenzoyl peroxide particles was further measured by the time for complete absence of the white granules that layered on the bottom of the plastic reaction container. Over the course of a day, results highly agreed with more exponential (R^2^ = .9962) electron-transfer relationships rather than linear (R^2^ = .7564) diffusion chemistry when lowering the cross-linking aldehyde concentration downward from 50wt% to 20wt % acrolein, Fig. 10. However, at just 10wt% acrolein some of the dibenzoyl peroxide granules were still undissolved and with no acrolein most of the granules were undissolved. So, the acrolein plays an additional role beyond crosslinking between lipid C=C double bonds and does indeed influence outer-shell valence free-radical generation through peroxide crystal bond decomposition.

**Fig. 11:**
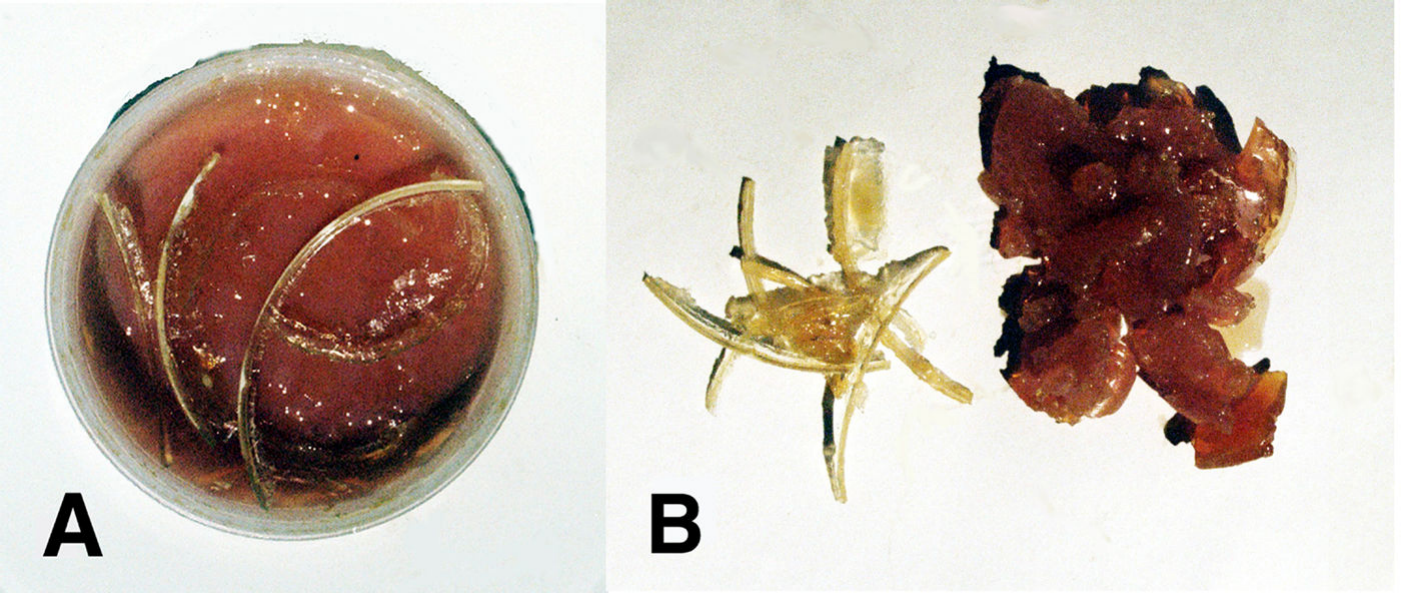
A–B. Lipid:Acrolein 50:50 mixture. (A) Polyethylene plastic vessel of diameter approximately 80 mm with lipid oil, acrolein and redox couple that produced a thermoset reactive secondary sequence solid gel on the bottom. The reactive secondary sequence mixture further crosslinked with oxygen to produce the lipid peroxidation crystalline material when exposed to atmospheric oxygen simultaneously in contact with the insulating plastic container seen on top after being physically dislodged from the vessel sides. (B) Lipid and acrolein products with peroxy crosslinking of the lipid peroxidation crystalline substance at 7.5 wt% on the left and reactive secondary sequence solid rubbery gel at 92.5 wt% on the right removed for mass analysis

For all acrolein concentrations from 10wt% to 50wt%, lipid peroxidation was noted by the appearance of a hard crystalline-like material that formed a solid curved surface oxidation which was particularly emphasized on the triple-interface of the polyethylene plastic reaction vessel (air/lipid/plastic). Photo images of the formed solids for the lipid:acrolein 50:50 mixture are presented in Figs. 11A–B.

The lipid peroxidation crystalline material at just 7.5 wt% of the total mass appears to occupy a disproportionate volume percentage when compared to the reactive secondary sequence rubbery-type solid gel at a much larger 92.5 wt% possibly due to oxygen crosslinking by extensive peroxy lipid termination (Equations 4–6) that has further trapped extra molecular oxygen. With acrolein available to produce easy vinyl-end free-radical crosslinking, reactive secondary sequence at some small scale may also possibly occur across the molecular oxygen O=O double bond similar to C=C crosslinking. Further note the differences in color between the yellow crystalline lipid peroxidation material and brown rubbery reactive secondary sequence lipid/acrolein thermoset polymer.

**Fig. 12:**
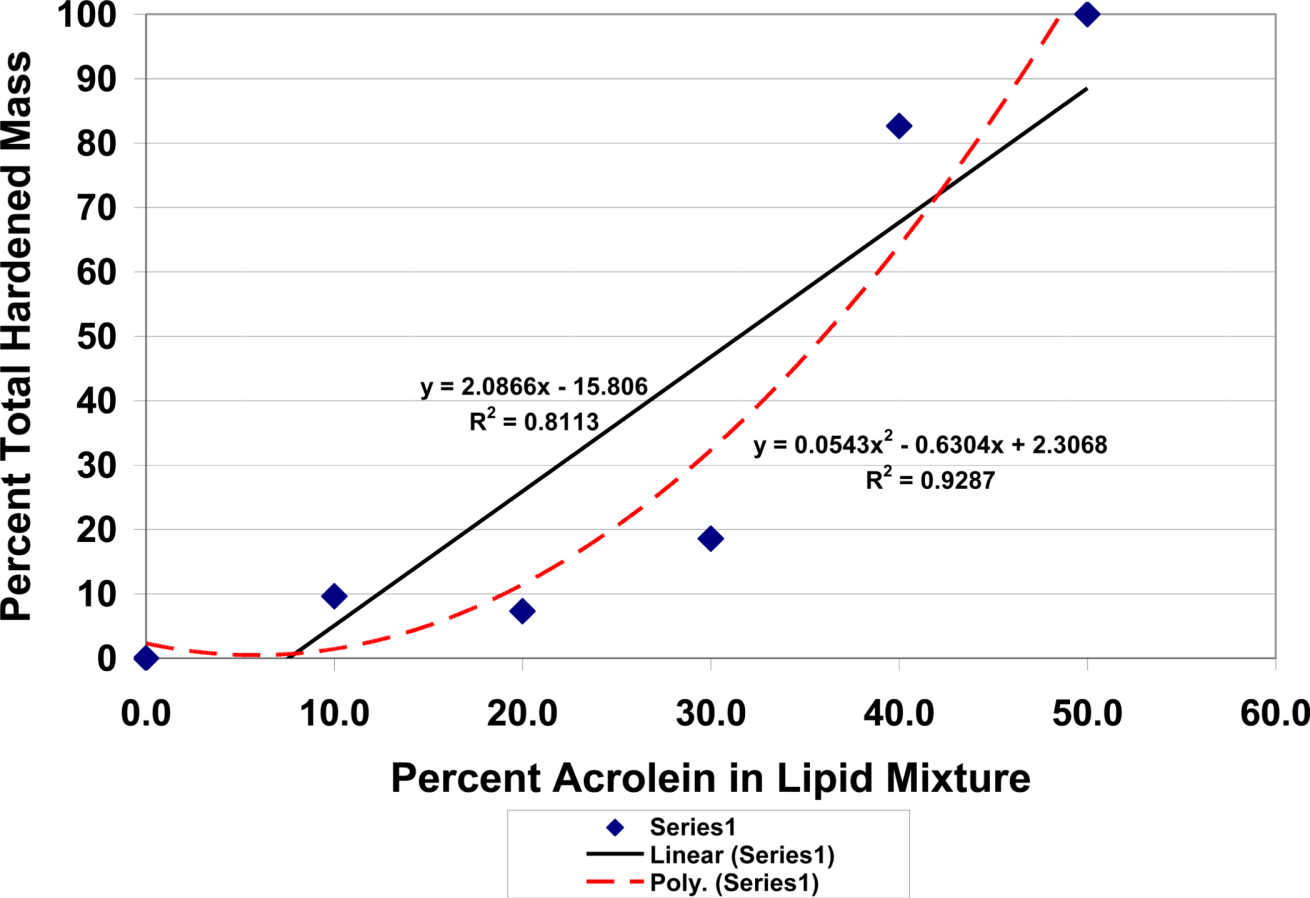
Increasing percent total hardened mass lipids with increasing acrolein concentration

Within the lipid:acrolein bulk, the solid reactive secondary sequence rubber-type adhesive gel-like mass that formed, Fig. 9, was most accentuated for lipid:acrolein ratios of 50:50 and 60:40, where lower molecular-weight fractions could phase separate from the solid as an adhesive lipid chain-lengthened reaction glue product. The lower-molecular weight lipid reaction products would squeeze toward the surface producing a glue-type adhesive medium that did not crystallize from molecular oxygen when not in contact with the plastic vessel. Related phase separation has been studied extensively for resin where lower molecular-weight crosslinking monomer is removed from the initial microgellation during chain-growth polymerization shrinkage as new bonds are formed [80, 83, 84]. When the percent total hardened mass from both crystalline substance and solid rubbery-type material were plotted against the concentrations of acrolein from 0% to 50%, although an electron-transfer exponential relationship could not be calculated, a second-order polynomial (R^2^ = .9287) could explain more of the data than a linear association (R^2^ = .8113), Fig. 12. Lipid peroxidation resulted at all acrolein concentrations from 10–50%, but the reactive secondary sequence solid thermoset polymer occurred only at 40–50% acrolein. Also, adhesive “gluey” crosslinked polymer fluids were produced at high concentrations from 10–40% acrolein.

At three months duration, even though percent total hardened mass and percent hardened gel were greatest at 50% acrolein, percent crystallized lipid peroxidation substance was highest at just 30% acrolein, Table 3. The interplay of molecular oxygen crosslinking with high diffusion from the surface may function to generate the irregular percentages of different substances produced. As a possible explanation, molecular oxygen is both influenced to crosslink as gellation starts and conversely prevented from diffusing through progressively increasing crosslinked chain-lengthening lipid:acrolein mixtures over time that might clarify the lack of association between acrolein concentration with crystalline lipid peroxidation material. Surface crosslink oxidation has previously been studied in rubber and silicone that similarly reduces oxygen diffusion into the bulk material [21–24]. Oxidation of rubber has even been shown to influence oxidative cleavage chain scission to produce carbonyls [24] that might further help explain the lack of association between possible acrolein crosslinking and hard lipid peroxidation crystalline substance, Table 3. Further, oxygen can sequester free radicals by a process known as oxygen inhibition to reduce C=C crosslinking and chain lengthening [4, 18, 25, 26] to produce oxide radicals that form glues [18, 26]. The formation of the “gluey” lipid fluids that reduce development of the solid rubbery-type material thereby increases the complexity of the overall reaction products.

Over an extended approximate period of one year during sustained reactive secondary sequence chain growth, the solid rubbery-type material continued to harden and stiffen while the gluey fluid increased in viscosity substantially. Upon heating the rubbery-type reactive secondary sequence solid material generally would harden indicative of a thermoset material, but would further release minor amounts of unreacted oil. With heating the lipid peroxidation crystalline material would become harder and darken without melting at much higher temperature which specifies a thermoset material with irreversible state of hardness [3, 4, 13, 14]. Heating improved the cure states to produce harder, stronger and stiffer materials of higher modulus for both materials. Increased viscosity or hardening and stiffening of a polymer over increasing time are further indicative of increased chain-growth polymer crosslinking [2–4, 13, 14, 27, 34–37] that produces a more durable and less soluble product [1–3].

### 3.2 Lipid Peroxidation Crystals Related to Nonpolar Polymer/Oxygen Sequestering of Free Radicals

Peroxyl recombination termination products of the acrolein chain-lengthened lipid oil from molecular oxygen may accentuate when free radicals are trapped by plastic insulation along the sides of the polyethylene container to heighten an electron-transfer environment. Polyethylene is a saturated polymer composed entirely of σ single bonds that do not provide electron mobility whereas unsaturated π C=C bonds impart electron delocalized flow [2]. An insulating interface that prevents electrons from escaping should help concentrate free radicals to optimize lipid alkene C=C double bond conversion to single bonds by reactive secondary sequence. In addition, molecular oxygen is a nonpolar molecule [5] that could accumulate by similar intermolecular forces of attraction [85] alongside of the nonpolar polyethylene container with peroxyl recombination termination products and increase the confinement of free radicals. As free-radical concentrations increase, electron transfer to form O_2_^−^˙ superoxide anion for crosslinking is highly expected to produce the crystalline lipid peroxidation material seen from the inside walls, Fig. 11. Because of the high affinity by electrons for oxygen and known excellent nonpolar molecular oxygen diffusivity through the nonpolar biologic lipid membranes [5], a large percent of the lipid free radicals reasonably near the atmospheric molecular oxygen interface should ultimately add peroxyl substituent groups. Oxygen molecular swing by two bond rotations attached to a lipid hydrocarbon and low spatial steric hindrance with small molecular size, Fig. 2, then provides opportunities for crosslinking lipids at more difficult C=C positions and also termination recombination products to make the crystalline lipid peroxidation substance produced along the plastic container walls at the surface. Free-radical concentrations may be sufficiently high when trapped by the lipid glue alongside the polyethylene insulation that O=O double-bond crosslinking by reactive secondary sequence may also have occurred. The broad similarities between the hard crystal-type triple interface for [atmospheric oxygen]/[unsaturated-lipid-acrolein-gel]/[nonpolar-plastic] when compared to [blood-oxygen with iron redox metal]/[unsaturated-lipids]/[nonpolar endothelial-vessel-cell-membrane-walls with nonpolar saturated fats] in close molecular proximity are therefore considered in some relative comparable form for a strong atherosclerotic plaque buildup and rapid vessel occlusion.

### 3.3 Related “Gluey” Adhesive Lipid Structural Risk Factors for Atherosclerosis

High levels of gluey adhesive materials from the experiments, note Table 3, that can harden into sticky solid products from lipid oil chain-growth reactive secondary sequence polymerization even with low acrolein concentrations provide new insight to atherosclerotic initiating factors at the vessel wall with endothelium cells. The adhesive properties of the gluey fluids and also growing sticky lipid solid polymer produce a situation where blood cell adhesion, protein binding, sclerotic fiber buildup and even microbial attachment become added serious possibilities on the arterial wall as initiating injuries. In addition to the progressive thermoset reactive secondary sequence adhesive solid and gluey polymer structures formed with oxide lipid peroxidation material from the original lipid oil with time, the exponent and second-order relationships shown in Figs. 10 and 12 emphasize the possible serious nature of free-radical pathology and especially with acrolein buildup where end stages may suddenly accelerate the disease process.

### 3.4 Free-Radical Polymerization Cure Shrinkage Measurement Test

When 71% lipid oil was combined with 28% acrolein and only 0.5% benzoyl peroxide and 0.5% cobalt naphthenate, after a day percent cured shrinkage measured 15.0% ± 1.7. As a result, another 90:10 oleic:linoleic lipid oil/acrolein model with lower concentrations of Fenton metal free-radical agents was demonstrated using cure shrinkage measurement of a solution rather than formed mass of several different solids. Also, with polymerization cure shrinkage testing, a more sensitive measure of lipid thermoset polymerization by chain-growth crosslinking can be performed rather than relying on mass measurements of formed solids. Further, increasing the concentrations of benzoyl peroxide and cobalt naphthenate slightly increased polymerization shrinkage percentages and increased overall solution viscosities.

### 3.5 Chain-Growth Polymerization of Vitamin A and Beta Carotene

**Fig. 13:**
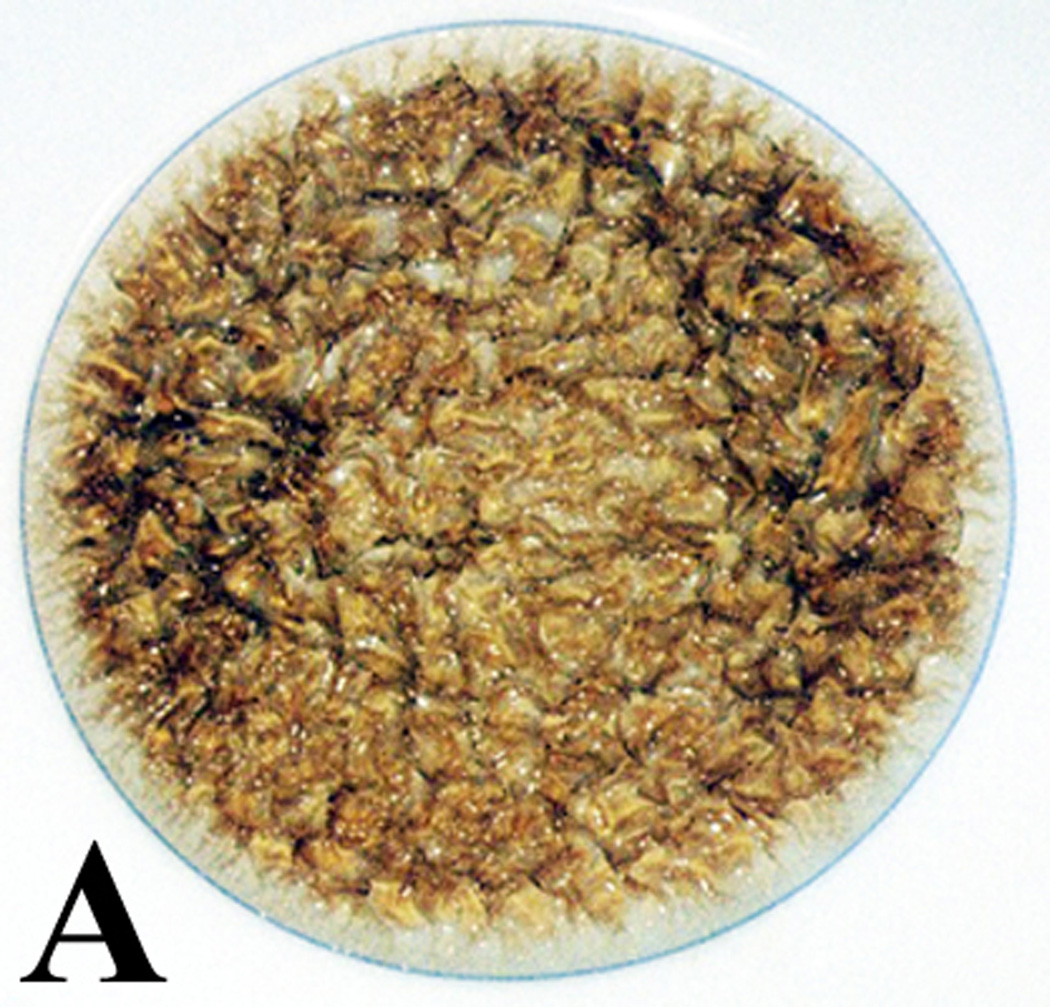

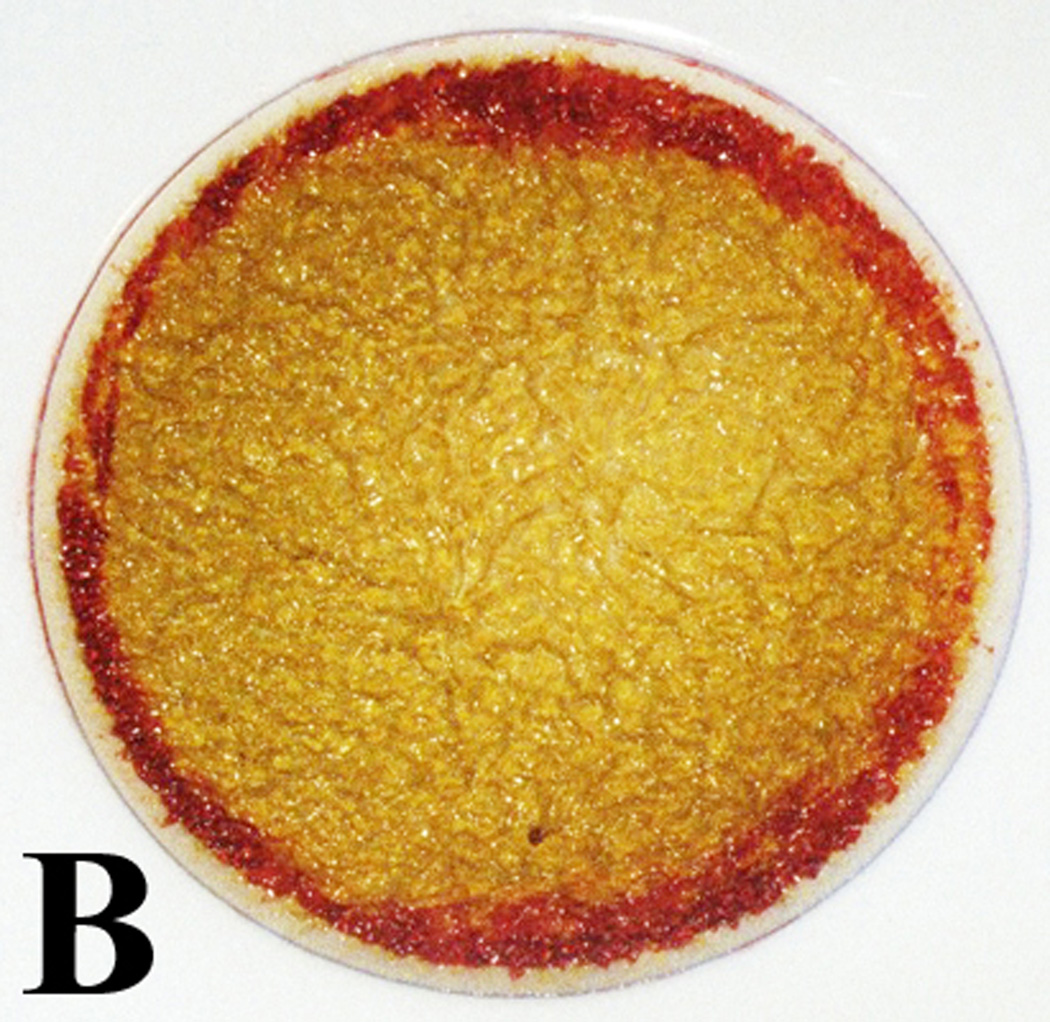

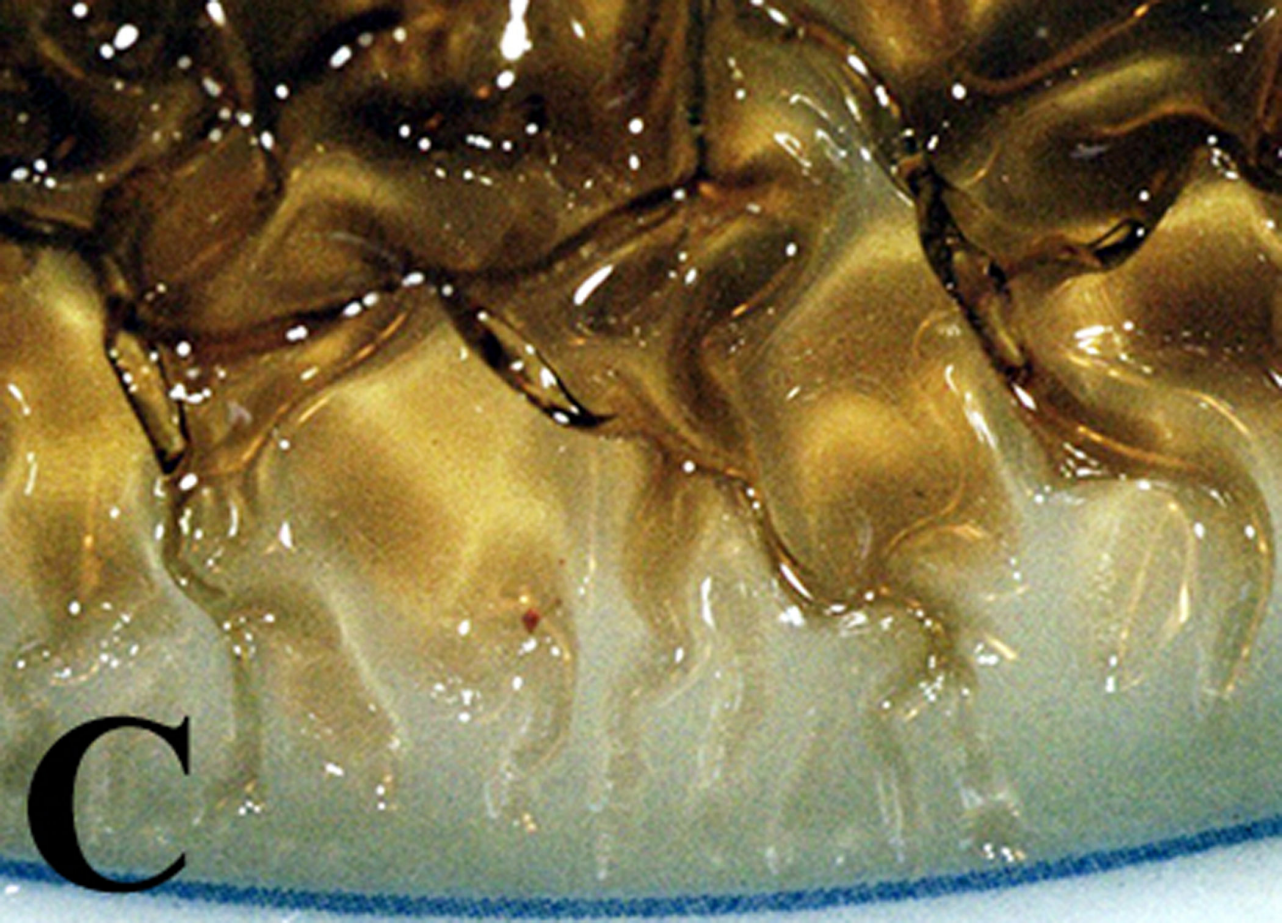

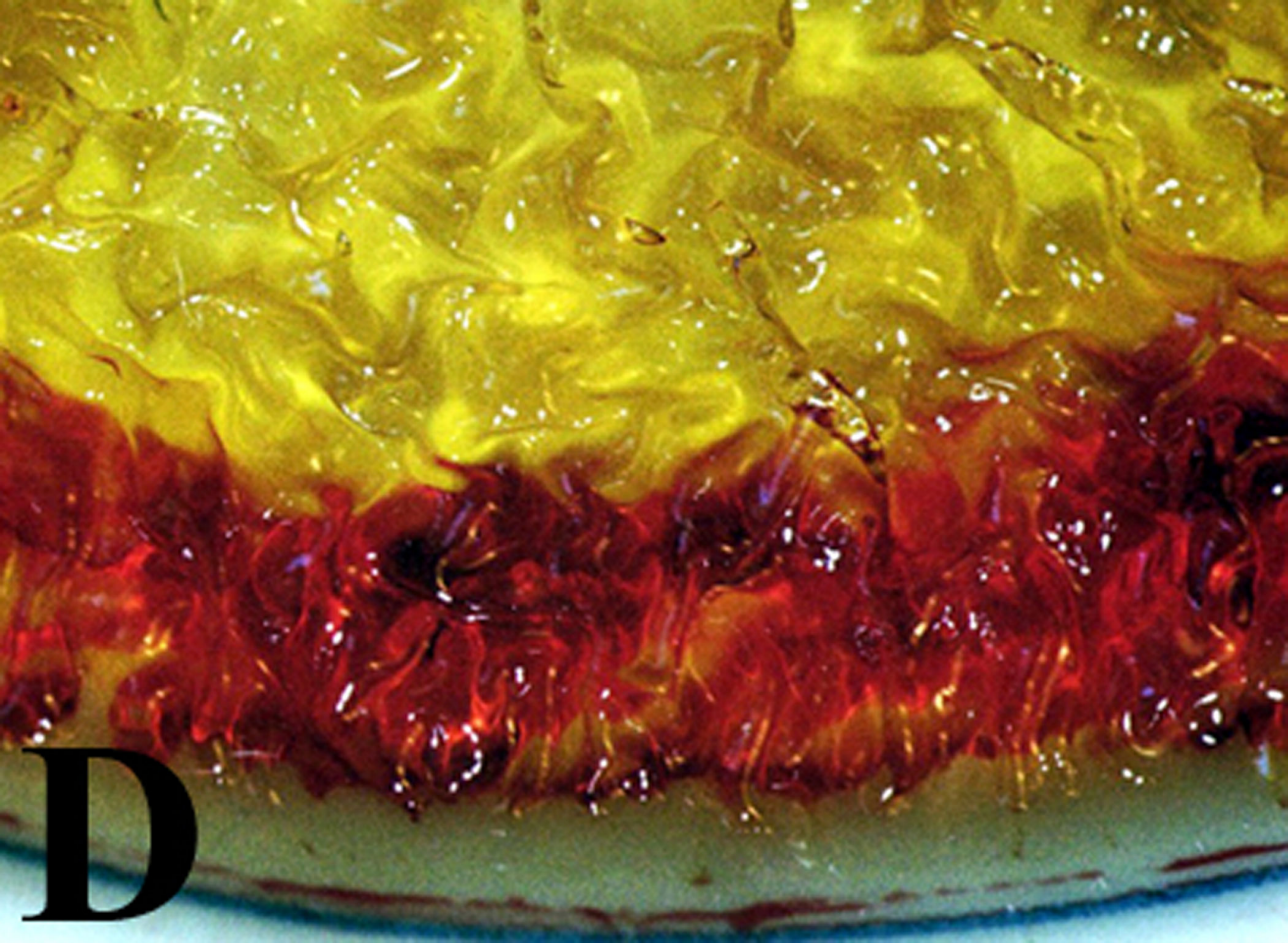

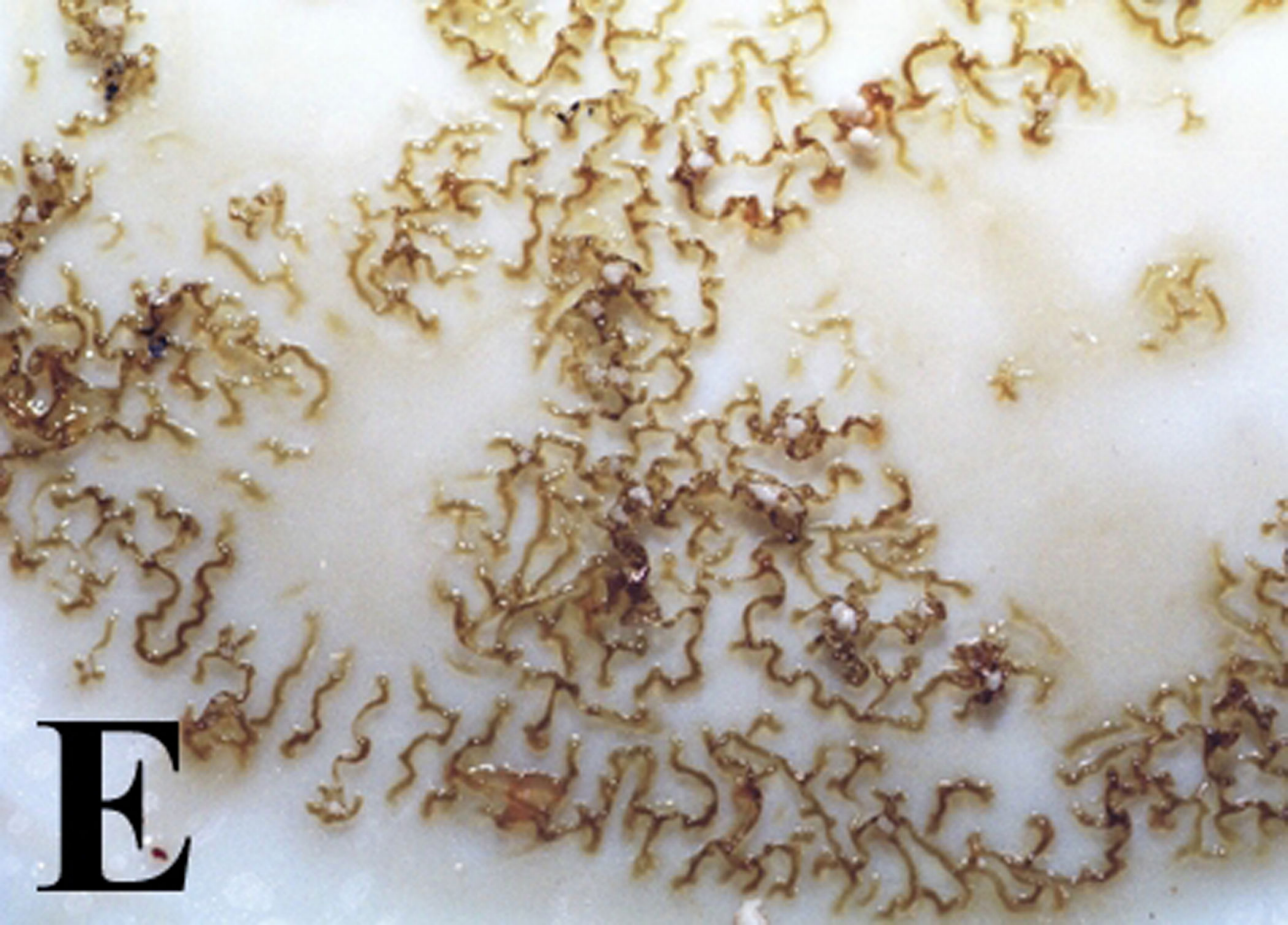

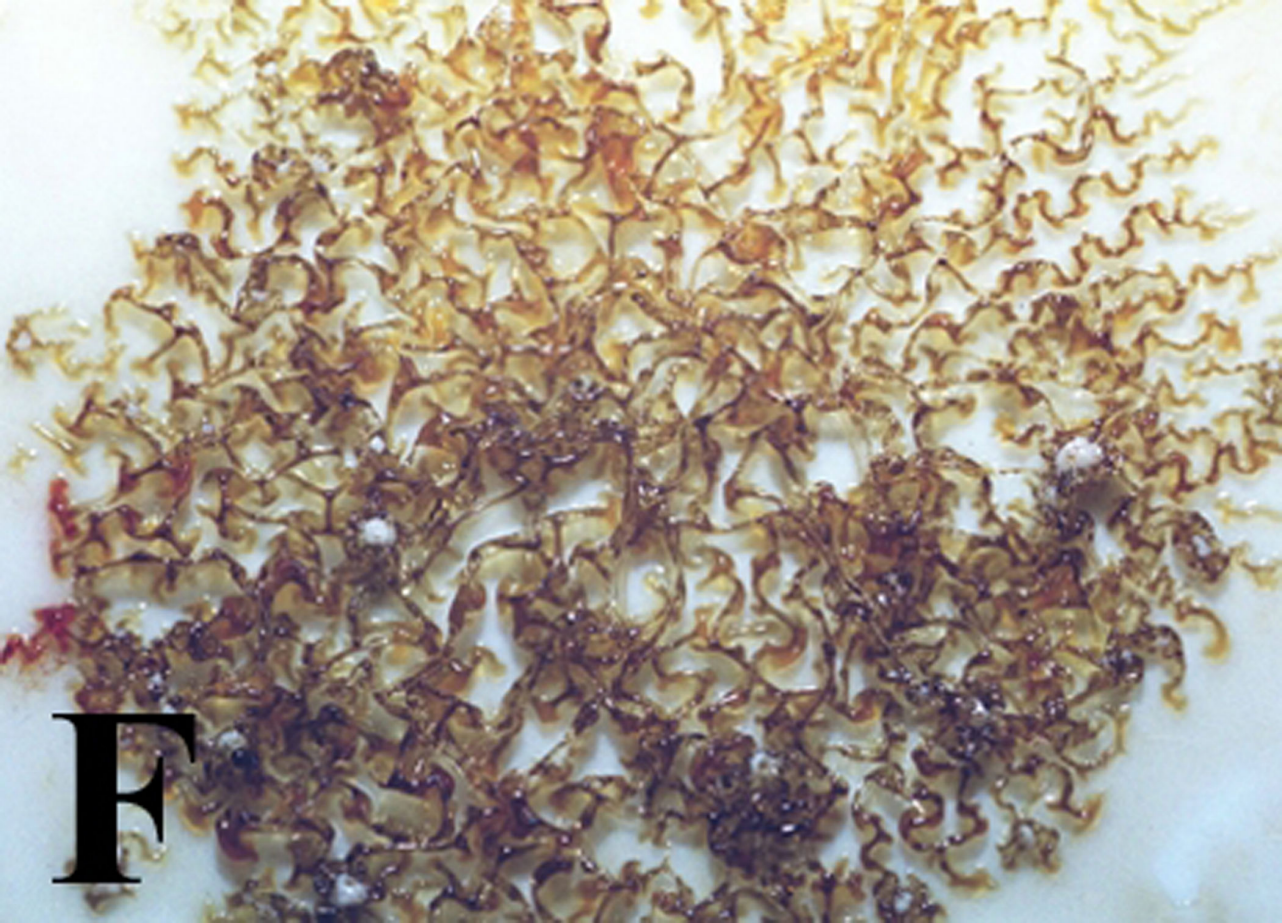
(A) vitamin A polymer gel low magnification (B) β,β-carotene polymer gel low magnification (C) vitamin A polymer gel higher magnification (D) β,β-carotene polymer gel higher magnification. (E) vitamin A polymer gel high magnification for thin film (F) β,β-carotene polymer gel high magnification for thin film. Thin films in E and F were exposed to higher concentrations of air oxygen that increased the free-radical peroxidation crosslinking and produced extensive cure shrinkage gaps between the polymers formed

When 4% cobalt naphthenate and 3% dibenzoyl peroxide were added slowly over a 3 day period to both low-viscosity unsaturated oils containing either vitamin A or β,β-carotene, viscosity increased to a point where both antioxidant-oil solutions began to gel. Elimination of all liquid flow occurred after 4 days for the vitamin A oil system and 9 days for the β,β-carotene oil system. Over time polymerization cure shrinkage was extensive noted by Fig. 13A–D photos at 12 days with the outer circumference rings as references for the original oil levels. Further, both antioxidant free-radical chain-growth thermoset polymers could be characterized as solid adhesive gels with slightly “sticky” surfaces. Free-radical polymerization cure shrinkage has gelled the low-viscosity unsaturated oils for vitamin A and β,β-carotene into adhesives that stick to the surfaces below and drawn the reactive secondary sequence thermoset polymer down further to crosslink polymer chains together and form solid irregular raised networks. The polymer surface levels of both antioxidants have dropped from the initial depth levels of approximately 4 mm and inward from the 9-mm diameter outer circular rings when examining Figs. 13 A and C for vitamin A or Figs. 13 B and D for β,β-carotene. By approximate polymerization shrinkage measurements, a 62% and 67% drop for vitamin A and β,β-carotene respectively in the original liquid levels has occurred as the polymers formed from the initial surfaces that started at the outer circular ring. Since the β,β-carotene adsorbs blue light a deep red coloration occurs that phase separated away from the larger oil base toward the circumference after 5 days. Also, overt macroscopic crystalline lipid peroxidation seen experimentally by using acrolein with linoleic and oleic fatty acids in Fig. 11 and measured through Table 3 was not noted for vitamin A or β,β-carotene free-radical thermoset polymerization. Thin films about 0.5 mm deep of both vitamin A and β,β-carotene with cobalt naphthenate and dibenzoyl peroxide free-radical redox agents subject to high concentrations for atmospheric molecular oxygen diffusion polymerized with volumetric shrinkage by reactive secondary sequence chain growth to separate and produce raised ribbons as ruffling patterns, Fig. 13E–F, that were much harder, stronger and stiffer than materials formed in the disks approximately 4.0 mm deep shown in Figs. 13A–D. Therefore, simple peroxidation crosslinking not apparent as bulk pure yellow crystals previously produced by the lipids mixed with acrolein has probably occurred on the molecular level toward the surface of the gelled reactive secondary sequence chain growth vitamin A and β,β-carotene polymers.

As an example of conjugated C=C double bonds posing a problem with antioxidants, the β,β-carotene analogue for vitamin A was identified in a large smoker study with 29,133 men to form an increased risk for lung cancer and total mortality [86]. The higher death risk was a result of numerous varied pathologies other than lung cancer such as coronary heart disease, stroke, hypertensive heart disease, cardiomyopathy, and aortic rupture [86]. In terms of a possible common mechanism to explain such diverse death risk, conjugated π-system carotenoids that act as antioxidants have previously been considered as a source for C=C addition reactions by free radicals delocalized over the entire conjugated system [48]. Further, from experimental evidence demonstrated earlier with Figs. 13A–F, vitamin A and β,β-carotene in unsaturated lipid-oil carriers can indeed produce reactive secondary sequence thermoset chain-growth polymers material that further increase structure by molecular oxygen crosslinking. Therefore, the risk involving a multiple C=C double-bond antioxidant vitamin of high molecular weight becoming over-saturated with delocalized electrons during variable induction phases to participate as a pool for free radicals and available C=C double bonds for reactive secondary sequence atherosclerotic-like chain-growth polymerization should not be ignored. Subsequent risk factors discovered with vitamin A and β,β-carotene supplementation would be especially elevated for people exposed to high levels of free-radical contaminants that could include cigarette smoke into lungs and cardiovasculature.

### 3.6 Antioxidant Comparisons for Hydroquinone with Vitamin E by Free-Radical Polymerization Shrinkage

**Fig. 14:**
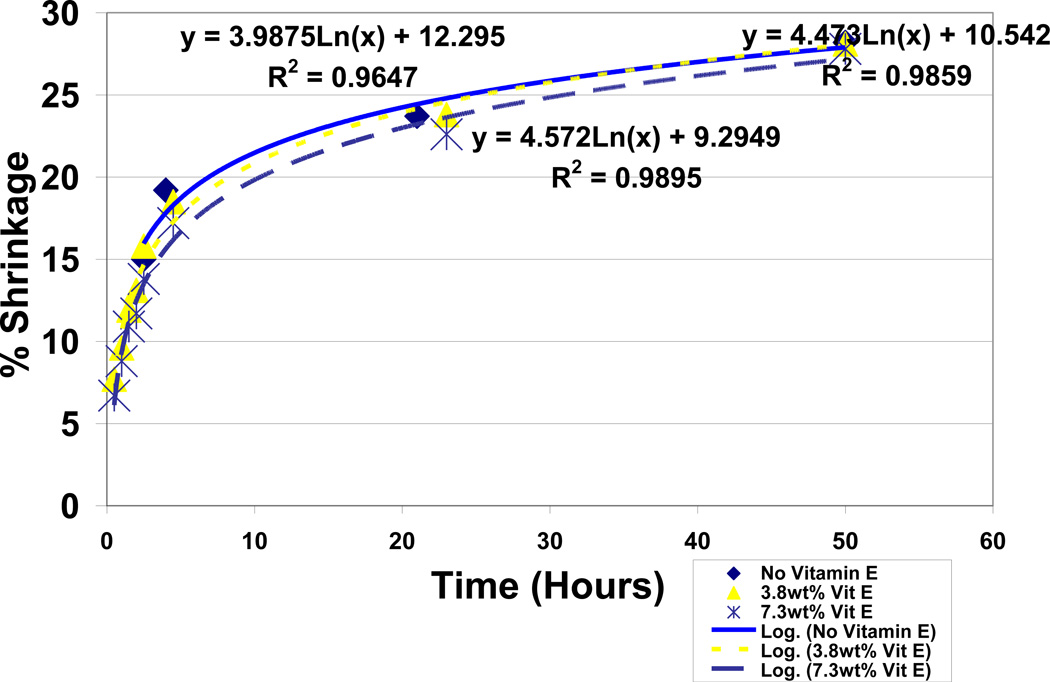
Lipid and acrolein polymerization shrinkage with vitamin E

**Fig. 15:**
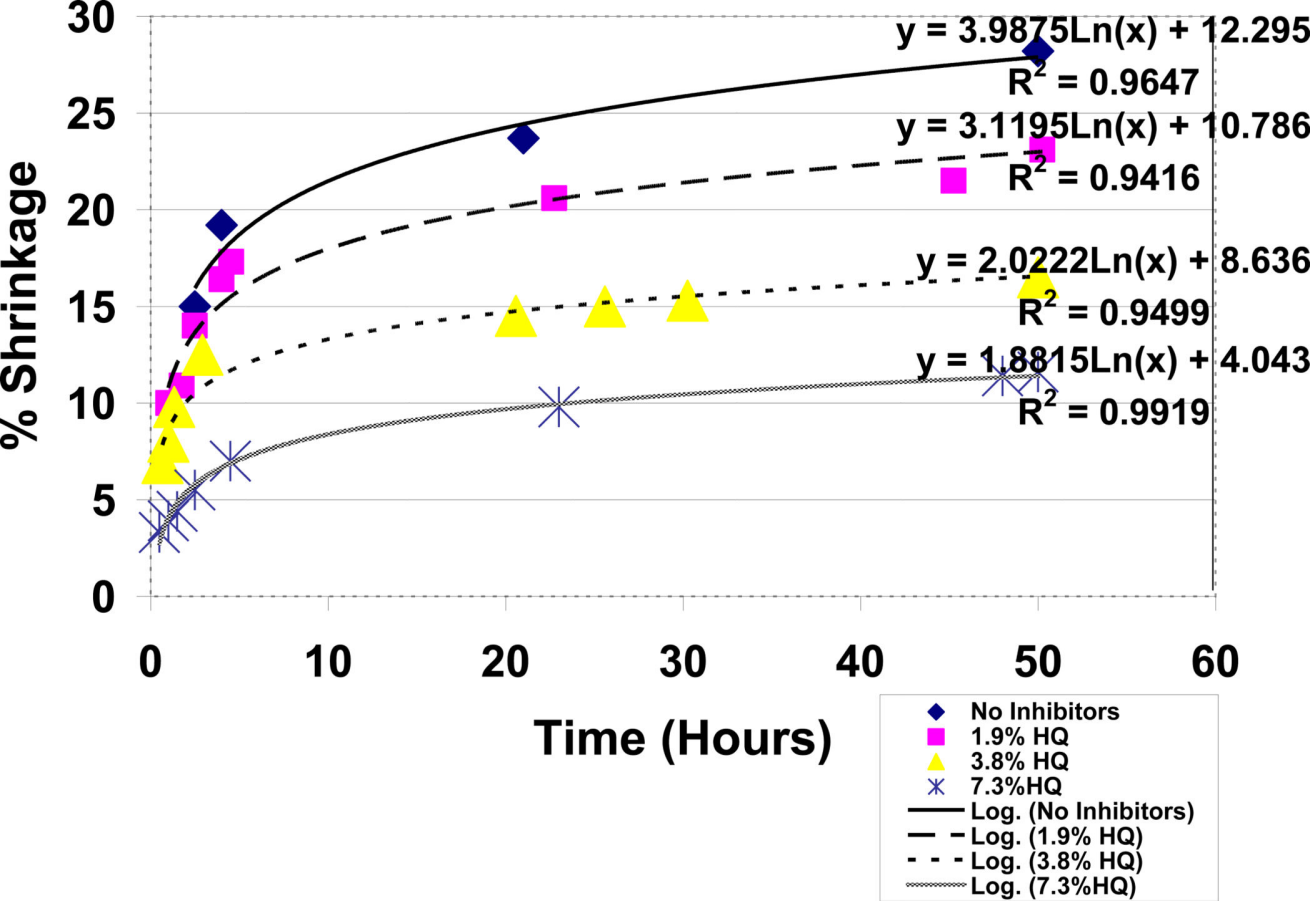
Lipid and acrolein polymerization shrinkage with hydroquinone (HQ)

**Fig. 16:**
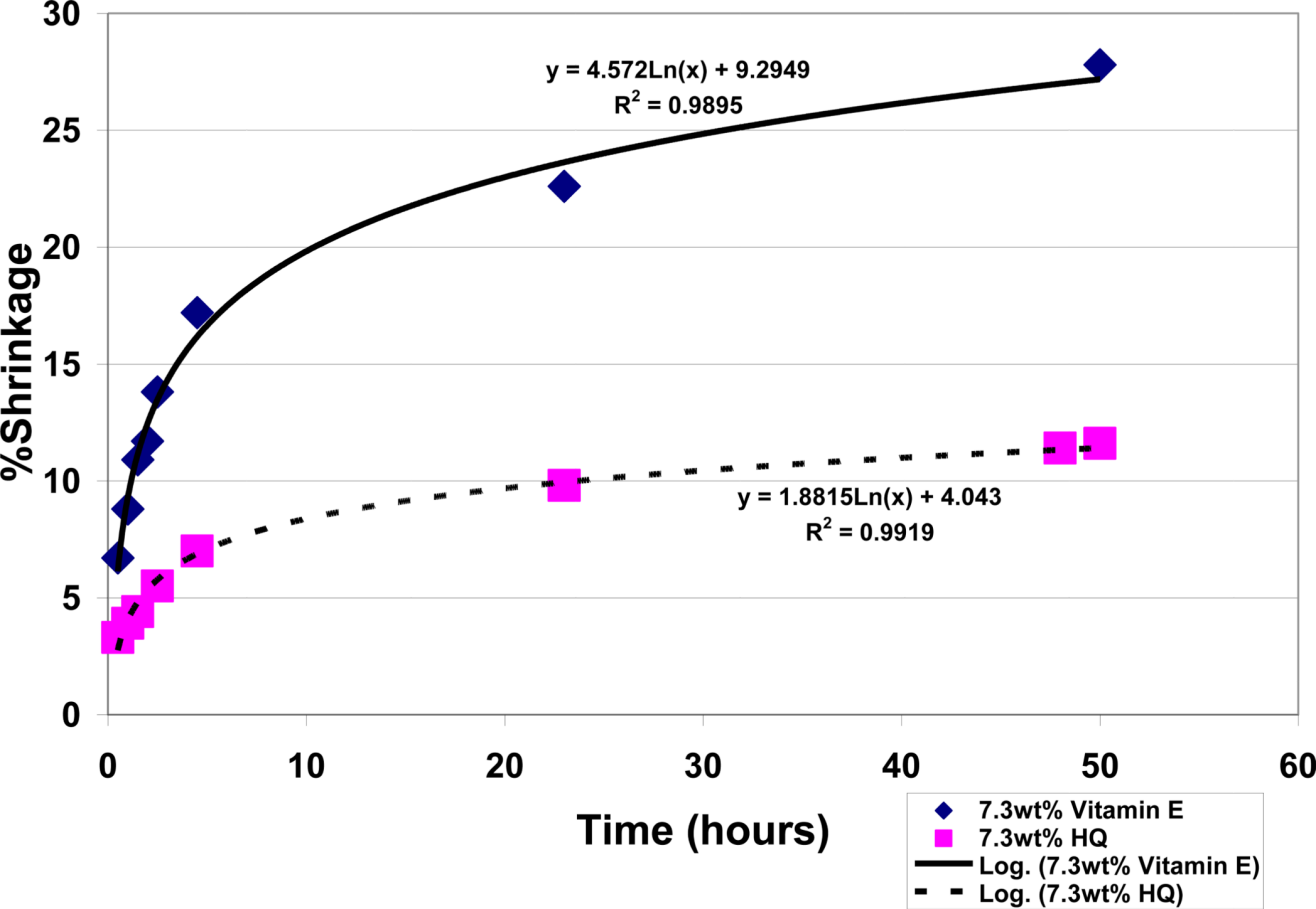
Lipid and acrolein polymerization shrinkage comparing 7.3% hydroquinone (HQ) with 7.3% vitamin E

Polymerization shrinkage testing for antioxidant free-radical inhibition over a 50 hour period with the current unsaturated lipid:acrolein model at a 50:50 ratio and the Fenton cobalt naphthenate and benzoyl peroxide redox couples at 4wt% each for making free radicals showed a dominating improvement in free-radical inhibition for hydroquinone over vitamin E. Vitamin E demonstrated no appreciable antioxidant properties without any significant reduction in polymerization during 50 hour test periods up to 7.3wt%, Fig. 14, and appears by experimental observation to possibly act as a lipid viscosity reducer. On the other hand, hydroquinone showed impressive antioxidant properties for removing free radicals with reductions in polymerization shrinkage during the 50 hour test periods from the 28.2% baseline at 0.0wt% down to 11.6% at 7.3wt%, Fig. 15. Hydroquinone and vitamin E are also compared simultaneously at 7.3wt% each in Fig. 16 up to 50 hours yielding significant statistical differences at all time periods with differences exaggerating continuously over time for reduced free-radical shrinkage produced by hydroquinone, Table 4. Percent shrinkage differences between hydroquinone and vitamin E with 7.3wt% each increased dramatically over time measured at 0.5 hours as 3.3 ± 1.6 for hydroquinone and 6.7 ± 2.3 for vitamin E (*P* = .05) while at 50 hours was 11.6% ± 1.3 for hydroquinone and 27.8% ± 2.2 for vitamin E (*P* =.00001). Polymerization shrinkage testing at 3.8wt% antioxidants each provided no significant statistical difference at 0.5 hours with shrinkage percents measured at 6.9% ± 2.2 for hydroquinone and 7.9% ± 0.5 for vitamin E but nevertheless still yielded a remarkable significant statistical difference at 50 hours with shrinkage percent values of 16.5% ± 4.3 for hydroquinone and 28.1% ± 2.6 for vitamin E (*P* = .01). Coefficient of Determination or R^2^ values for hydroquinone groups ranged from a low of .9416 to a high of .9919 by log relationships with time to explain most of the variability for percent shrinkage. Vitamin E groups similarly produced high R^2^ values from .9647 to .9895 to explain variability.

Others have questioned the ability of alpha tocopherol vitamin E to act as an antioxidant [70]. Although *in vitro* activity for vitamin E can be demonstrated with hydroxyl radicals, *in vivo* proof is not easily found or supported [70, 87]. In addition, vitamin E cannot scavenge hydroxyl free radicals at the same high level as DNA, proteins or lipids [70]. In the reactive secondary sequence polymerization study presented now, the unsaturated lipids with oleic 90% and linoleic 10% further provide vulnerable C=C pi bonds for free radicals to easily attack and be trapped when compared to vitamin E with low antioxidant properties.

Over a prolonged period of approximately two years with all styrene vials capped and each group sealed in separate closed polyethylene containers to exclude a source of atmospheric oxygen, all test groups continued to crosslink and increase free-radical polymerization shrinkage values at relatively similar rates. However, vitamin E started to show some antioxidant properties with reduced polymerization shrinkage from the control with no antioxidants. In addition, an unusual property regarding the consistency or flow of each group could be characterized as developing extreme differences. The control group, without any vitamin E or hydroquinone, polymerized into sticky reactive secondary sequence thermoset solid products that also formed a type of strong hard adhesive lipid peroxidation on the surface walls of the styrene vial containers. On the other hand, all groups with vitamin E or hydroquinone maintained the lipids in an oily fluid state. Although the vitamin E groups continued to crosslink lipids at higher levels than the same hydroquinone groups, the vitamin E viscosities were noticeably lower than similar hydroquinone groups. With low antioxidant free-radical inhibiting properties especially when compared to hydroquinone, but with superior lubricating properties to improve lipid fluidity suggests that alternate mechanisms for vitamin E may occur to account for *in vivo* success. Vitamin E has previously been considered a weak antioxidant [70] and appears to have non-antioxidant functions [71]. The oxidation product of vitamin E has never been found while activity is measured by hydrogen-atom donation [69]. Further, membrane fluidity of erythrocytes has been shown to decrease during lipid peroxidation with concurrent diminished levels of polyunsaturated acids and vitamin E [68]. Although related reactive secondary sequence free-radical crosslinking of lipid chains will also decrease the fluidity for fatty acids oleic and linoleic, some points are being considered regarding the possibility of vitamin E providing some special types of nonantioxidant related lubricating properties to protect membrane fluidity and the polyunsaturated fatty acids [72]. Hydroquinone would possibly also provide similar types of protection regarding membrane fluidity over time.

### 3.7 Marcus Theory Exponential Relationships [88]

When comparing outer-shell electron transfer between the relatively small molecular weight reactants for benzoyl peroxide decomposition as a function of acrolein, an exponential rate predicted by Marcus Theory is found true compared to a linear regression, Fig. 10. With the exponential regression obtaining an R^2^ value of .9962, acrolein concentration could explain almost 100% of the variability for time during decomposition of the benzoyl peroxide crystals. Polymerization shrinkage as a measure of covalent bond formation with decreasing free-radical reactants similarly followed Marcus Theory predicted by natural logarithmic rates in Figs. 14 and 15 plotted without any vitamin E or hydroquinone respectively with an R^2^ = .9647. Although vitamin E antioxidant properties were minimal and did not make an appreciable difference in the lipid/acrolein free-radical polymerization shrinkage, nevertheless electron transfer relationships over time for covalent bond formations with decreasing free-radical reactants were maintained as predicted with natural log Equations by Marcus Theory, Fig. 14, with R^2^ = .9859 at 3.8wt% and R^2^ = .9895 at 7.3wt%. Inhibition of lipid/acrolein polymerization by hydroquinone free-radical inhibitor continues to provide similar natural log relationships, Fig. 15, with R^2^ = .9416 at 1.9wt%, R^2^ = .9499 at 3.8wt% and R^2^ = .9919 at 7.3wt%.

### 3.8 Reactive Secondary Sequence Polymerization and Lipid Peroxidation in Pathology

Organic hydrocarbons derived from primeval geologic petroleum or synthetic biological sources and engineered for exothermic free-radical crosslinking thermoset polymer materials should be expected to contain some levels of ambient-physiologic free-radical chemistry similar to tissues investigated medically on a pathophysiologic basis. Such thermoset polymers have in fact been studied at the most advanced intensive levels in the development of structural Aeronautical/Aerospace polymer-matrix composite materials [46, 47]. By incorporating the basics for addition alkene chain-growth Organic Chemistry [2, 3] with reactive secondary sequence crosslink free-radical thermoset Polymer Science [1–4, 13, 14, 18, 26] and mitochondrial produced free-radical/acid availability [5, 11, 29], a mechanistic explanation of the primary downstream lipid oxidation events should be improved upon to better model many extreme biocomplex Medical conditions. Physiologic conditions following oxygen depletion during cell metabolism by mitochondria include both build up of free radicals and also lower pH [5, 11, 29]. Following chronic free-radical buildup with oxidative cleavage at lower pH to produce shorter reactive alkenes especially as acrolein breakdown products develop, reactive secondary sequence C=C chain-growth polymerization with crosslinking might reveal possible influential chemistry that develops a loose interpenetrating network of lipids through other molecules to alter fluidity of normal structures. In terms of lipid peroxidation, surface oxidation by molecular oxygen is well-recognized for alkene C=C double-bond addition and recombination for chain lengthening that further interferes with oxygen diffusion into the deeper bulk material [21–24]. Conversely, in order to explain biocomplexity, molecular oxygen also produces free-radical inhibition during chain lengthening of thermoset polymer-cure systems by accepting an electron [4, 18, 25, 26] so that the O_2_^−^˙ superoxide anion is much less reactive than the free-radical particle waveform. Also, as larger molecules chain lengthen and crosslink by covalent bonding, the possibility of trapping other small molecules, long molecular chains or even cells increases to further prevent common bioflow pathways.

Atherosclerotic biocomplex lipid-rich-core plaque progression related to oxidative stress by free radicals might be better explained with alkene chemistry and reactive secondary sequence chain-growth interpenetrating networks as persistent chemistries develop. Extracellular lipid “fatty streak” deposition is the first sign of atherosclerosis in coronaries for children and young adults [5, 6, 8]. In the progression of atherosclerosis, endothelial oxidative stress is associated with free-radicals and reactive oxygen species [5, 30, 89, 90], LDLs [3, 89, 90], ischemia [90], inflammation [3, 90, 91], and infection [3, 90, 91]. Free radicals in turn develop during periods of ischemia at the mitochondrial level [3, 11, 29] that further promote atherosclerosis [9, 50, 92]. Further, free radicals oxidize LDLs that build up in vessel walls [3, 89], increase by neutrophiles during inflammation [93] and appear during infection [92, 94, 95]. Free radicals also accumulate throughout all layers of the atherosclerotic wall [89]. Thicker fibrotic lipid-rich-core plaques subsequently interfere with oxygen diffusion [3] that would accelerate the formation of mitochondrial free-radical pathology by ischemia [3]. Interference with oxygen transport at some level that could create both excess mitochondrial free radicals and acids might then be better acknowledged through the progressive accumulation of lipid pathological states at all stages.

**Fig. 17:**
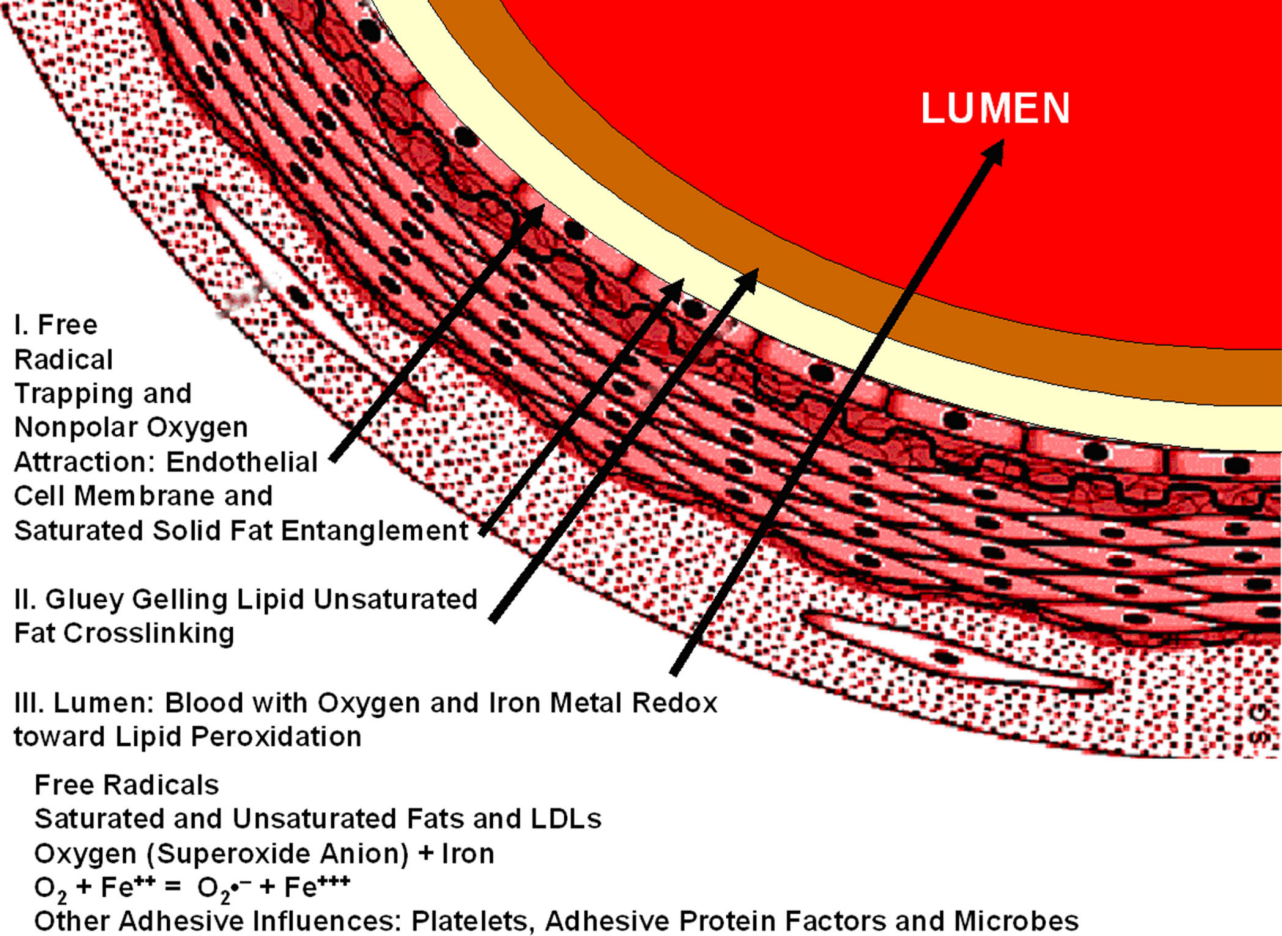
Initiating Triple Interface for lipid peroxidation includes: (I) Nonpolar cell membrane with nonpolar saturated lipids from animal fats that can insulate to trap high concentrations of free radicals and also attract nonpolar molecular oxygen. (II) Unsaturated Lipids and acrolein for crosslinking by Reactive Secondary Sequence into a gluey gellation stage to further combine with oxygen. (III) Blood Oxygen and most other molecular species that combine to initiate atherosclerosis

As free radicals crosslink alkenes, oxygen diffusion is reduced even more to deeper layers [21–24]. Subsequent lipid peroxyl free-radical recombination termination products, Equations 4 and 6, that produce a hard lipid peroxidation crystalline-like substance are of particular concern at the previously noted triple interface for [oxygen]/[lipid-acrolein-gel]/[insulating-polymer] or the postulated [blood-oxygen]/[crosslinked-lipid]/[arterial-wall], Fig. 17. As hard lipid peroxidation crystal formation occurs even at the molecular level, subsequent oxygen diffusion to deeper layers would then be restricted. Saturation of a hydrocarbon polymer with all σ bonds interferes greatly with electron mobility and further imparts nonpolar chemistry [2, 3], that needs some appreciation with similarities by intermolecular forces of attraction [85]. Highly diffusive nonpolar molecular oxygen recombination termination could exaggerate at a nonpolar insulating interface where solid nonpolar saturated fatty acids may accumulate alongside the tissues of nonpolar endothelial lipid cell membranes in an artery to further produce some type of potential nonpolar free-radical insulation trapping. Increasing free-radical concentrations then provide the possible reaction conditions for both combined lipid double-bond molecular oxygen peroxidation into crystal structure, Fig. 2 and Fig. 11A–B, also with lipid alkene C=C reactive secondary sequence crosslinking, Fig. 3, into a solid lipid gel-like polymer, Fig. 9A and Fig. 11A–B.

**Fig. 18:**
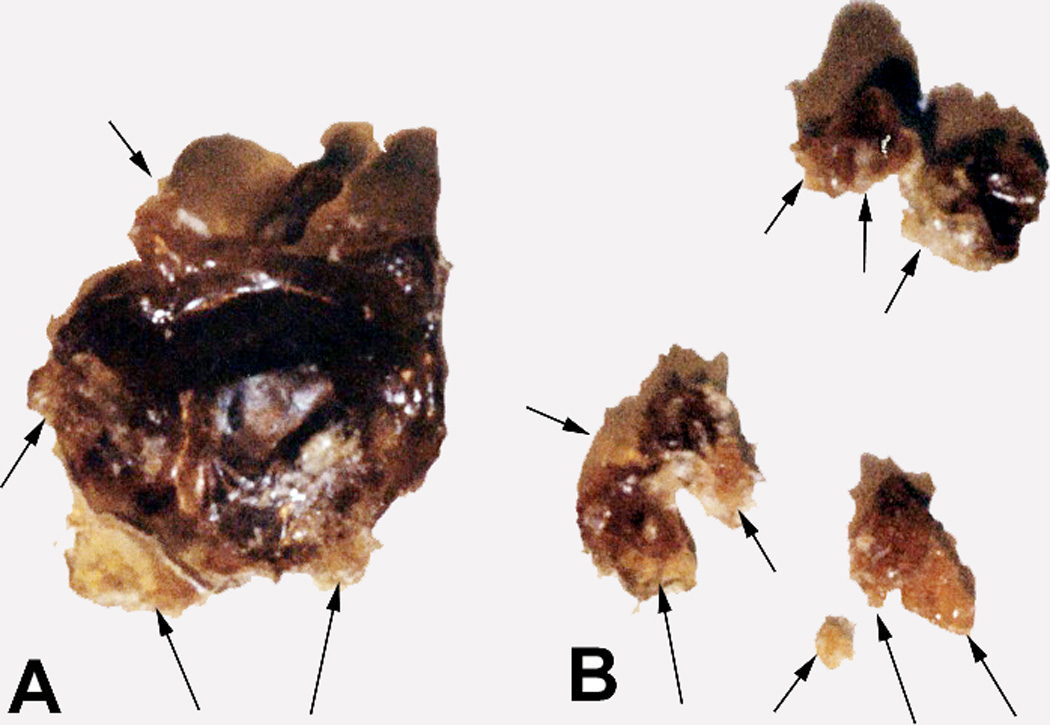
A–B. Wound surface hard outer layers with margins displaying lipid peroxidation crystalline materials corresponding to interfaces with borders of nonpolar/healthy tissues

As a much less complex comparative peroxidation substance, the gellation wound surface material photographed in Fig. 18A does in fact demonstrate crystalline lipid-like material at several margins of the outer edges (arrows) and would correspond to an interface with an insulating border of nonpolar healthy tissue. Fig. 18B provides several additional lipid peroxidation wound surface borders (arrows) that would have similarly been in contact at an interface with nonpolar insulating healthy tissues. In addition, from the experimental results presented here, the low-molecular-weight acrolein aldehyde lipid breakdown product with a vinyl C=C end group and an organometallic species appear to be commonly found biofactor reactants that exist for initially forming a solid lipid gel mass and other adhesive products from unsaturated lipids to produce the crystalline lipid peroxidation with surface molecular oxygen. Subsequent solid gelled polymers, reactive gluey products and crystalline lipid peroxidation material create potential devastating molecular interpenetrating vascular occlusive consequences with saturated lipid fats. If chain lengthening occurs as postulated by some type of exponential or second-order electron-transfer relationship, then atherosclerotic-related problems diagnosed and treated at the earliest possible time should have greater prognosis for healing. In fact, as previously mentioned Marcus Theory for outer-shell electron transfer does indeed predict exponential rates [88] that correspond to increasing free-radical concentrations in pathological conditions.

Ischemic cell death associated with formation of arachidonic acid metabolic breakdown products and free radicals produce a wide range of damaging cellular effects that include complete plasma cell membrane depolarization [96]. Extensive lipid peroxidation is also associated with cell membrane depolarization [30]. Membrane depolarization consequently can be reversed by treatment with free-radical scavengers that includes oxygen reperfusion and use of antioxidant vitamin E [90]. Since vitamin E does not appear to greatly diminish free-radical crosslinking by antioxidant properties presented in Figs. 14 and 16, cell recovery to reduce membrane depolarization is possibly due to an alternate property of vitamin E noted by experimental observation to improve fluidity of fatty acids such as oleic and linoleic, Fig. 5, by a lubricating property to reduce lipid-chain entanglements. Cholesterol with a steroid ring-system structure is thought to maintain cell membrane fluidity [2, 5, 11], but might be expected not to compatibilize with phospholipids molecules such as oleic or linoleic fatty acids through intermolecular forces of attraction as well as vitamin E with a similar hydrocarbon tail structure, Fig. 8. In fact, vitamin E has been shown to associate in cell membranes with saturated fatty acids that are further found expended of cholesterol [72]. Further, cholesterol forms solid crystals as early signs of atherosclerosis [97] whereas vitamin E is highly viscous oil and also cosmetic moisturizing agent that maintains lubricant capacity to flow at body temperature [98]. Consequently, vitamin E might reduce lipid entanglements of the cell membrane through a new form of health benefit so the cell could possibly be better able to retain negatively charged molecular species with more uniform membrane insulation with fewer gaps and hold an acceptable negative voltage potential. The movement of superoxide anion radicals from mitochondrial membranes has been shown to reduce at high vitamin E concentrations in a complex non-antioxidant role [99]. Although C=C bonds could continue to crosslink by reactive secondary sequence free-radical mechanisms, unsaturated lipid chains more capable of being drawn together to entangle and help form membrane gaps are possibly reduced sufficiently by vitamin E to produce cellular health observed experimentally. In effect, lipid membranes would subsequently maintain more pliable electrical insulation structure with vitamin E rather than becoming rigid and entangled with molecular gaps that would be able to more easily release negatively charged free radicals during free-radical chain-growth C=C crosslinking. Membrane depolarization is manifested by the resting prepotential to be more accessible to the threshold potential for quicker sudden action potential spike [3, 11], that would increase firing for pain and would thus appear to be an important major loss of cell function that could be relevant to many forms of pathology especially including possible cardiac arrhythmias.

Changes in plasma cell membrane morphology [100] over time by C=C lipid free-radical polymerization shrinkage crosslinks and with free-radical covalent binding to DNA bases, chromatin condensation, DNA cleavage or DNA-protein crosslinks [95, 101] might be able to explain pleomorphism in cancer associated with free radicals. In turn, pleomorphic changes in cell shape, size and nuclear/cytoplasm ratios [95, 100], are associated with both cancer and free radicals [102–104]. Further, ion loss is one of the most commonly reported apoptotic events [105]. So, a partial mechanistic explanation for one important apoptotic event may be due to C=C crosslinked plasma cell membrane gap shrinkage or channels formed as phospholipid fatty ester chains are drawn together/pulled apart in addition to C=C oxidative cleavage lipid breakdown to corresponding aldehydes. Cell death related to free radicals has further been shown to form highly reactive short chain aldehydes considered a direct lipid oxidation product responsible for diabetic pancreatic beta cell destruction [52].

Many cancer tumors are associated with infection [92, 94], and also correlated with chronic inflammation [95, 106], where successful treatment can include use of anti-inflammatories [106]. Inflammation in turn is highly linked to the formation of free radicals from hydrogen peroxide [93, 95]. Carcinogenesis is further linked to low oxygen concentrations [95, 102–104]. With regard to the low oxygen levels during hypoxia, mitochondrial cellular energy synthesis requires oxygen as the final electron acceptor to combine with hydronium or H_3_O^+^ to produce eventual water as the end product [5, 11, 29]. Electrons formed concurrently with hydronium during mitochondrial respiration metabolism are in the lowest energy state when transferred to oxygen [5, 11 29]. Without oxygen, acids must accumulate with hydronium as free electrons form. Hypoxic states or anoxia that lower the pH would then be directly associated with free-radical oxidant production and consequently possible lipid oxidative unsaturated alkene cleavage aldehyde products. Shorter aldehydes that still retain C=C groups such as acrolein then accelerate progressive reactive secondary sequence lipid chain lengthening. Hypoxia is already accepted as a general condition that promotes tumors [103, 104], supports cancer recurrence [104], intensifies malignancy [103, 104], increases metastases [102, 104], and inhibits chemo/radio therapies [104].

If in fact free-radical energy is sufficient for overall carbon:hydrogen bond dissociations postulated by the current lipid peroxidation theory, Equations 1–3 [15, 16, 19], presumably aided by a concentration enzyme effect with free radicals and acids, carbon:carbon bond dissociations that would produce alkyl radicals should be considered as well. The carbon:carbon bond is longer for easier access and requires less energy for dissociation than the carbon:hydrogen bond, Table 1 [2, 28]. Subsequent alkyl or carbon centered free radicals are highly reactive with the methyl free radical (H_3_C˙) being the smallest and least stable [2, 3]. Regarding possible methyl free-radical recombination with DNA, alkylating agents were identified early as mutagens and direct carcinogens in 1969 [107, 108]. Since then, methylation of DNA bases is now considered a probable cause for cell mutation and carcinogenesis [109]. Of special interest, covalent binding by methyl radicals to DNA at cytosine bases inactivate genes that in particular are thought to suppress cancer [95]. Even further, cancer cells often have chromosome defects with pieces translocated in the wrong place and extra or missing chromosomes [95, 110], which might be explained through biocomplexities involving excessive mitochondrial ischemia that can produce both molecular breakdown and recombination as two separate possible consequences from the excess production of both acid and free radicals. As an additional characterization of electron pair bond dissociation, atomic bond stretching has been shown to produce free radicals with alkenes such as isoprene [111]. Since the optimum C-C single bond and C=C double bond lengths are on the order of only about 154 and 134 picometers respectively [1], tensile deformed and stretched tissue states seen with sudden aneurysms, tumors, boils or other fast growing tissue pathologies might produce free radicals mechanically independent of biochemistry to exacerbate pathology.

Acknowledgement of free-radical reactive secondary sequence C=C chemistry could provide new insight into possible initiating events before major biocomplexities evolve into gross pathology. With an improved awareness of the initial free-radical pathobiocomplexities, preventive measures to eliminate free-radical sources that promote thermoset reactive secondary sequencing or dangerous oxidative cleavage of alkenes at lower pH levels might be more closely monitored. The possible intense bonding by molecular oxygen with high diffusivity between already gelling lipids from acrolein and a peroxide-organometallic redox couple into a rapid growing hardened thermoset lipid peroxidation crystalline material appears to be another concern with atherosclerosis and also other medical conditions related to oxygen reperfusion injury [30, 112] or possibly opaque tissue formation associated with oxygen toxicity [11]. Crosslinking by molecular oxygen at the cellular level may further influence free radicals and acids produced even by limiting oxygen diffusion to mitochondria at deep levels closer to the nucleus. Consequent better recognition regarding the seriousness of free-radical saturation into tissues may then be cause for sufficient alarm to encourage preventive measures that eliminate exposure at the various sources and also develop nutrition with therapeutic treatment countermeasures.

### 3.9 Future Lipid Work through Polymer Science

An appreciation for lipid chain-growth reactive secondary sequence C=C π double-bond conversion to σ single bonds should help in determining initiating events upstream of the ensuing pathobiocomplexities that develop structural pathology. In particular, lipid lubricant properties that change during macromolecular chain-growth to adhesive solid materials would appear capable of intermingling with saturated fats and further trapping proteins and cells in atherosclerosis. As a result of understanding thermoset reactive secondary sequence alkene and especially vinyl chemistry, better mechanistic free-radical pathobiological models should be formed. The initiating events regarding free-radical polymerization or inhibition can then be studied under highly controlled laboratory test situations already established through Chemistry and Polymer Science. An improved understanding of the initiating species could further be pivotal in reducing related Medical conditions subsequently studied at a biological degree. The credibility of Polymer Science should encourage adjunct antioxidant therapy studies to prevent formation of lipid chain-growth polymers through a greater understanding of the highly active and purified free-radical inhibitors and retarders currently in use by the polymer industry.

## 4. CONCLUSIONS

A standardized textbook hypothesis for lipid pathobiologies during oxidative states is proposed from basic Organic Chemistry and also from free-radical-cure thermoset chain-growth Polymer Science. Two common alkene C=C double bond reactions that encompass addition reactive secondary sequence thermoset chain growth and oxidative breakdown should be acknowledged when evaluating lipid pathobiologies. In terms of structural pathology, the outer C=C π orbital will readily combine with a free radical to form a sigma bond with one carbon atom and form a new free radical on the alternate carbon atom. Chain reactions though numerous C=C double bonds can then extensively create macromolecular structure by repeated reactive secondary sequence chain-growth. At body temperature, the unsaturated lipid C=C reactive secondary sequence can proceed with no energy input by simply contacting a sufficiently concentrated free-radical source when C=C vinyl end groups are available for crosslinking. Conversely, regarding breakdown chemistry, under highly concentrated oxidant free-radical conditions and especially with combined acidic lower pH levels, a lipid C=C bond can break down into two aldehydes. As smaller molecular weight aldehydes are formed and especially smaller aldehydes that still contain end-group C=C vinyl double bonds subsequent reactive secondary sequence cross-linking of multiple adjacent lipid alkene planar C=C bonds becomes more favorable. The biological proteins known as enzymes also provide even more favorable molecular thermodynamic mixing for the formation of aldehydes such as the highly reactive vinyl lipid breakdown product acrolein. Therefore, biocomplexities commonly observed during pathological states might be better explained by anoxia/hypoxia oxidative stress during cellular mitochondrial metabolic energy synthesis where oxygen is needed to both accept electrons and then also combine with acid to produce water as the final waste product. Molecular oxygen normally inhibits crosslinking at lower free-radical concentrations, but may also crosslink unsaturated lipids at higher free-radical concentrations particularly with the lipid breakdown product acrolein. On the other hand, as a controversial discrepancy, the original saturated tetrahedral alkane-type -CH_2_- methylane lipid peroxidation model requiring carbon:hydrogen single bond dissociation is not energetically favorable without an enzyme or acid and proposed chain-lengthening events are minimal. During atherosclerosis pathology, normal unsaturated alkene lipid oils would associate with saturated fat/oil entanglement by similarity of chemistry through intermolecular forces of attraction, but in addition provide interpenetrating free-radical pathways to permanently crosslink C=C double bonds for structural chain lengthening. The interplay of molecular oxygen as an electron acceptor and diffusive molecule that can both inhibit chain lengthening at lower concentrations and also crosslink lipids by recombination to terminate reactive secondary sequence chain lengthening introduces biocomplex events. In terms of structural pathology, molecular oxygen can even form hardened crystalline-like lipid peroxidation substances following development of a gel phase from free-radical chain-lengthened lipids using reactive acrolein and a peroxide/metal redox couple. Such oxygen lipid crosslink chemistry may further even include small forms of reactive secondary sequence polymerization addition across the O=O double bond at an unsaturated lipid interface similar to the more extensive bulk thermoset C=C crosslinking. Superoxide anion radicals, as O^•^_2_^−^, associated with ischemic reperfusion injury might likewise crosslink to create structure and possibly even interfere with oxygen diffusion and blood flow. Therefore, oxygen should be carefully evaluated during the course of treatment for any serious medical procedure with antioxidant therapy to ensure that the many potential dangerous consequences of anoxia/hypoxia related free radicals formed during cellular mitochondrial respiration metabolism are generally avoided. Due to the numerous lipid biocomplexities identified with competing reactions that occur relative to normal feedback homeostasis, Medical treatments should be reevaluated carefully and often to consider alternatives when progress slows or side effects are encountered before more severe Medical pathologies possibly develop. Vitamin A and β,β-carotene produced bulk thermoset reactive secondary sequence polymerization using a Fenton redox couple in an unsaturated lipid medium thus supporting large-scale clinical trials that identified increased related mortality risks for cigarette smokers. In addition, hydroquinone proved to be a valuable future antioxidant with high potential as a Medical adjunct in testing that measured covalent solution shrinkage during reactive secondary sequencing when compared to the standard antioxidant vitamin E.

## Figures and Tables

**Table 1:** Bond Dissociation Energies for Homolytic Cleavages A:B → A∙ + ∙B

Single bond - (references)	Bond dissociation energy	
	kilocalories/mole	kilojoules/mole
1. CH_3_:H (A, B)	104–105	436–439
2. CH_3_CH_2_:H (A, B)	100–101	420–423
3. (CH_3_)_2_CH:H (A)	96	401
4. (CH_3_)_3_C:H (A)	91	381
5. C_6_H_5_:H (A, B)	111	464–465
6. CH_3_CH=CHCH_2_:H (B)	85.7	358.2
1. CH_3_:CH_3_ (A, B)	88	368
2. CH_3_CH_2_:CH_3_ (A)	85	355
3. (CH_3_)_2_CH:CH_3_ (A)	84	351
4. (CH_3_)_3_C:CH_3_ (A)	81	339
5. C_6_H_5_:CH_3_ (A, B)	79–102	317–427
6. CH_3_CH=CHCH_2_:CH_3_ (B)	72.9	305.0
HO:OH (A, B)	51	213
CH_3_O:OCH_3_ (B)	37.6	157.3
C_2_H_5_O:OC_2_H_5_ (B)	37.9	158.6
APP:P* (A, C)	7.3–7.4	30.5–31.0

Arabic numbers correspond to identical substituent groups for either :H or :CH_3_ single bonds.

A [2];

B [28];

C [29];

* APP:P (Adenosine triphosphate reaction with water to form adenosine diphosphate and hydrogen phosphate and hydronium ion as the standard energy currency for living organisms). kilocalorie = 4.1819 kilojoules at 20°C

**Table 2:** Lipid:Acrolein Free-Radical Shrinkage Model Comparing Vitamin E with Hydroquinone

Antioxidant	Weight Percent
None	0.0
Vitamin E	3.8
Vitamin E	7.3
Hydroquinone	1.9
Hydroquinone	3.8
Hydroquinone	7.3

**Table 3:** Percent Acrolein In Oleic/Linoleic Lipid Mixture Measuring Different Chain-Lengthening Substances Produced Over Three Month Period With 4wt% Dibenzoyl Peroxide/4wt% Cobalt Naphthenate Fenton Metal Redox System

Percent of Acrolein	50%	40%	30%	20%	10%	0%
**Type of Material**	**Percent of Total Mass**					
Solid Crystalline	7.5	1.3	18.6	7.3	2.0	0
Rubbery Solid Gel	92.5	81.4	0	0	7.6	0
Total Solid Material	100.0	82.7	18.6	7.3	9.6	0
Gluey Fluid	0	17.3	81.4	92.7	90.4	0
Clear Liquid Oil	0	0	0	0	0	100

**Table 4:** Figure 16 Comparisons for Hydroquinone and Vitamin E Differences by T-Tests with Unequal Variances

Time (Hours)	t Stat	*P* value
0.5	2.78	.05
1	5.32	.001
1.5	5.58	.001
2.5	7.91	.0001
4.5	6.06	.0001
23	14	.00001
50	14.5	.00001

## ABBREVIATIONS

C=C: carbon-carbon double bond
carbon:hydrogen: carbon-hydrogen single bond (dissociation)
oxygen:oxygen: oxygen-oxygen single bond (dissociation)
carbon:carbon: carbon-carbon single bond (dissociation)
RO:OR: peroxide bond
π: pi bond
σ: sigma bond
LDL: low density lipid
HDL: high density lipids
